# A pilot study assessing the clinical utility of deep learning-reconstructed 3D-echo-planar-imaging-based quantitative susceptibility mapping in multiple sclerosis

**DOI:** 10.3389/fnins.2025.1544376

**Published:** 2025-07-16

**Authors:** Dimitrios G. Gkotsoulias, Matthias Weigel, Alessandro Cagol, Nina de Oliveira Soares Siebenborn, Esther Ruberte, Josef Pfeuffer, Cristina Granziera

**Affiliations:** ^1^Translational Imaging in Neurology (ThINk) Basel, Department of Medicine and Biomedical Engineering, University Hospital Basel and University of Basel, Basel, Switzerland; ^2^Department of Neurology, University Hospital of Basel, Basel, Switzerland; ^3^Research Center for Clinical Neuroimmunology and Neuroscience Basel (RC2NB), University Hospital Basel and University of Basel, Basel, Switzerland; ^4^Division of Radiological Physics, Department of Radiology and Nuclear Medicine, University Hospital of Basel, Basel, Switzerland; ^5^Department of Health Sciences, University of Genova, Genova, Italy; ^6^Application Development, Siemens Healthineers AG, Erlangen, Germany

**Keywords:** quantitative susceptibility mapping (QSM), gradient-recalled-echo (GRE), 3-dimensionalsegmented-echo planar imaging (3DEPI), multiple sclerosis (MS), susceptibility (χ), central vein sign (CVS), paramagnetic rim lesion (PRL), artificial intelligence (AI)

## Abstract

**Background:**

Quantitative susceptibility mapping (QSM) has emerged as a promising paraclinical tool in multiple sclerosis (MS). This retrospective pilot study aims to evaluate whether a recently proposed deep learning-assisted, k-space-operating reconstruction, denoising and super-resolution technique (DLR) applied on 3D-echo-planar-imaging (3DEPI) protocols, has the potential to improve the quality and clinical utility of QSM in MS, at 3T. Secondarily, we assess whether applying DLR vs. a conventional reconstruction (CR) can improve the quality of QSM based on noise-susceptible, fast 3DEPI protocols.

**Methods:**

3T MRI 3DEPI-data were acquired on seven MS patients and offline-reconstructed using CR and DLR. *A sample size of 433 lesions was identified, based on* FLAIR segmentation. Two experts, independently and method-blinded, rated lesion-wise the CR- and DLR-3DEPI-derived QSM, assessing the confidence in identifying paramagnetic rim lesions (PRLs), central vein sign (CVS), QSM hyper/isointense lesions and image quality. Gradient-recalled-echo (GRE), 2- and 1-average 3DEPI (acquisition time: 7:02, 3:44, and 1:56 min, respectively) from a healthy individual were offline-reconstructed using CR and DLR. Derived QSM maps were compared visually and quantitatively.

**Results:**

Deep learning reconstruction-3DEPI-based QSM was rated significantly higher for the confidence in identification of the MS-specific biomarkers (hyper/isointense lesions: *P* < 0.001, CVS: *P* = 0.01) and overall image quality (*P* < 0.001), compared to CR-3DEPI-based. Inter-method agreement was high for both raters (Cohen’s κ = 0.98/0.92), suggesting that DLR improves the quality without changing the rater’s perception of the individual QSM-related clinical findings. Additionally, QSM derived from fast DLR-3DEPI with a fourfold acquisition-time reduction compared to GRE, exhibited excellent visual and quantitative consistency with GRE-based QSM.

**Conclusion:**

Our results constitute a first demonstration of the enhanced quality and clinical utility of the DLR-3DEPI-based QSM in MS.

## 1 Introduction

Quantitative susceptibility mapping (QSM) is an MRI-based modality, with which estimates of the voxel-wise bulk magnetic susceptibility are obtained (Wang et al., 2017; Deistung et al., 2017). The physical underpinnings of QSM contrast are associated with tissue molecular composition (especially with iron and myelin content) and disease-induced damage (Möller et al., 2019). In multiple sclerosis (MS), QSM enables the identification of pathological changes that play a crucial role in the pathophysiology of the disease. Unlike conventional MRI, QSM aids in characterizing and disentangling the microstructural changes accumulating within MS lesions (Granziera et al., 2021; Langkammer et al., 2013; Gillen et al., 2021; Kaunzner et al., 2019). It is increasingly employed to identify chronic active lesions, a subset of MS lesions characterized by persistent inflammatory and degenerative activity associated with a more aggressive disease course (Absinta et al., 2019; Cagol et al., 2024a; Bagnato et al., 2024). In these lesions, QSM can reveal the presence of a rim of paramagnetic signal along the lesion edge, indicating the accumulation of iron-laden macrophages and microglia (Huang et al., 2022; Dimov et al., 2022). These paramagnetic rim lesions (PRLs) represent promising diagnostic and prognostic biomarkers, and their importance in the management of MS is expected to grow in the near future (Preziosa et al., 2021; Martire et al., 2022). Additionally, QSM has recently emerged as a promising technique for differentiating MS lesions based on myelin content, providing biomarkers sensitive to remyelination. In fact, MS lesions exhibiting a hypointense or isointense QSM contrast have been shown to accurately identify remyelinated areas upon pathological examination (Rahmanzadeh et al., 2022). Furthermore, QSM can support the identification of the central vein sign (CVS), an MRI biomarker with high performance in supporting the differential diagnosis of MS (Castellaro et al., 2020; Cagol et al., 2024b).

In practice, gradient-recalled-echo (GRE) or 3D-segmented-echo planar imaging (3DEPI) acquisitions are often used as basis for the estimation of susceptibility (χ) maps (QSM Consensus Organization Committee et al., 2024). 3DEPI-based acquisitions are faster, a crucial factor for clinical applicability. However, the low SNR, increased susceptibility artifacts and geometric distortions typically provided by 3DEPI-based sequences has led to the recommendation of using GRE in clinical settings, despite its longer acquisition times (QSM Consensus Organization Committee et al., 2024; Tourell et al., 2024). Recently, a vendor-specific deep learning-assisted (U-net based), k-space-operating reconstruction, denoising and super-resolution technique (Siemens Healthineers, Erlangen, Germany) has been proposed (Hammernik et al., 2019; Lopez Schmidt et al., 2023; Behl, 2021). Initial assessments of the deep-learning reconstructed (DLR) MRI contrasts indicated excellent quality and increased SNR along with substantial reduction in scanning and reconstruction time (Pfeuffer et al., 2024) compared to conventional reconstruction (CR)-based maps. Given that resolution, artifact limitation, and SNR are crucial for enhancing tissue characterization, we aimed to assess the potential added value of the DLR pipeline in characterizing QSM-based biomarkers in people with MS.

To achieve that aim, in this pilot study, rapid 3DEPI acquisitions are combined with the DLR to investigate the clinical accuracy and validity of the susceptibility maps derived, in the context of MS. Toward that aim, 3T MRI 3DEPI data of seven MS patients were post-reconstructed using product CR and research DLR. The derived χ maps were assessed by two experts – blinded to the reconstruction method – who rated for each contrast lesion-wise the confidence in identifying the presence of PRLs, CVS, QSM hyperintense and isointense lesions, as well as the overall QSM image quality based on the perception of susceptibility/motion/other artifacts and SNR. Additionally, to assess the acquisition time (TA) advantage we can achieve using DLR, while maintaining the QSM quality, we acquired 3T GRE as well as 2- and single-average 3DEPI data (acquisition time: 7:02, 3:44, and 1:56 min, respectively) on a healthy individual. QSM maps from the progressively faster acquisitions were compared visually and quantitatively using the QSM derived from the GRE data as a reference.

## 2. Methods

### 2.1 MR imaging

Three-dimensional-segmented-echo planar imaging (Sati et al., 2014) MRI data (0.67 mm isotropic, TE: 35 ms, TR: 64 ms, flip angle: 10°, no GRAPPA, TA ∼6.5 min) from seven MS patients were obtained at the University Hospital using a Siemens MAGNETOM Prisma 3T system (Siemens Healthineers, Forchheim, Germany) equipped with the manufacturer’s 64-channel head/neck array coil. The patients were part of the INsIDER study that was approved by the local ethics committee (IRM of northwest Switzerland) and registered at clinicaltrials.gov (NCT05177523). Table 1 presents the cohort’s characteristics. FLuid Attenuated Inversion Recovery (FLAIR) images for each patient were also available. The patients were selected only based on providing a high lesion load and not based on any other pathological parameter.

**TABLE 1:** Multiple sclerosis cohort clinical information.

Patients with multiple sclerosis	Age (years)	Sex (M/F)	Disease phenotype	EDSS score	Disease duration (years)	T2LV (mm^2^)	Lesions count	Medication
Patient 1	56	M	PPMS	6	9.65	37,752	60	Ocrelizumab
Patient 2	23	F	RRMS	1	2.5	7,567	33	Ocrelizumab
Patient 3	63	F	PPMS	4	11.64	39,373	40	Rituximab
Patient 4	64	M	PPMS	5	3.88	4,003	68	Ocrelizumab
Patient 5	54	F	SPMS	6.5	20.82	13,427	89	Rituximab
Patient 6	42	M	RRMS	2	14.47	15,559	63	Dimethyl fumarate
Patient 7	22	M	RRMS	2	0.33	17,468	80	Ocrelizumab
	46 ± 17*	4/3	–	3**	9.0 ± 7.3*	–	61 ± 18.6*	–

Age is noted at the time of the MRI scan. EDSS, Expanded Disability Status Scale; T2LV, T2-lesion volume; PPMS, primary progressive MS; RRMS, relapsing-remitting MS; SPMS, secondary progressive MS. *Mean ± standard deviation in the entire population. **Median in the entire population.

Additionally, for the assessment of the potential time advantage we can obtain using DLR, one healthy participant (male, 32 years old) underwent a brain MR examination at the same scanner. An RF-spoiled GRE scan was obtained using a 3D Fast Low-Angle SHot (FLASH) sequence (Frahm et al., 1986) (0.7 mm × 0.7 mm × 2 mm, TE = 20 ms, TR = 39 ms, flip angle = 15°, GRAPPA Factor = 3, TA ∼7:02 min). 3DEPI (Sati et al., 2014) scans with two and one averages (3DEPI2 and 3DEPI1, respectively) were also obtained during the same session, with matching parameters to the GRE protocol (0.7 mm × 0.7 mm × 2 mm, TE = 20 ms, TR = 56 ms, flip angle = 21°, ETL = 5, TA ∼3:44 min and ∼1:56 min, respectively).

All scan parameters can be found in Supplementary Tables 1–3. All MR acquisitions were performed by MW (MR Physicist, 25 years of experience) and DGG (MRI/Neuroimaging researcher/Engineer, 5 years of experience).

### 2.2 CR and DLR-based magnitude/phase and QSM maps estimation

A dedicated system with GPU resources (8 GB) was used for the retrospective reconstruction of magnitude and phase images with both CR and DLR. CR refers to the vendor-implemented, common reconstruction methodology for obtaining magnitude and phase images from the MR-system. The DLR methodology accepts as input the k-space raw data (in a vendor-specific format, as.dat file) and comprises two independent, sequential processing steps. Firstly, the deep resolve boost (DRB) step (Hammernik et al., 2018): images are generated on the acquired resolution using a variational network architecture with six iterations that alternate between parallel imaging reconstruction and 3D image regularizations using U-nets [with L1 loss function and Adam (Kingma and Jimmy, 2014) as optimizer]. The network parameters were determined through supervised training based on training data derived by ∼500 fully sampled 3D head, abdomen, and pelvis datasets, from healthy volunteers, acquired at 1.5T and 3T MRI scanners. Secondly, the deep resolve sharp (DRS) step (Afat et al., 2022; Chaika et al., 2023; Almansour et al., 2021): the obtained images from DRB were interpolated using a deep learning-based super-resolution algorithm with a factor-of-two interpolation. Both steps were implemented in PyTorch (Paszke et al., 2019) trained on a dedicated GPU cluster and with networks exported for prospective use in the scanner reconstruction pipeline. It is worth noting that the training data did not include any of the data assessed in the current study. In both CR and DLR, “Adaptive Coil Combination” from the vendor-supplied inline reconstruction was used, as suggested by the most recent QSM Consensus (QSM Consensus Organization Committee et al., 2024).

Quantitative susceptibility maps were estimated from the reconstructed phase images using CUDA-implemented total generalized variation (TGV-QSM) algorithm (Langkammer et al., 2015) which combines in a single optimization problem all the major QSM pipeline steps (i.e., the phase unwrapping, background field removal, and field-to-source inversion). The specific TGV implementation used is described in Stewart et al. (2022), along with all relevant parameterization details. The number of iterations was set to 2,500, for the dipole inversion step and regularization factors were α_1_: 20 and α_2_: 30. QSM reconstruction was performed on the same system as the reconstruction, taking ∼4 min for the control data and ∼15 min for the MS patients, due to the interpolated submillimeter resolution. A schematic representation of the processing is depicted in Supplementary Figure 1.

### 2.3 Qualitative analysis of MS clinical characteristics on QSM maps

Quantitative susceptibility mapping does not constitute a generic tool for lesions identification but provides highly specific information useful for the further characterization of microstructural changes in lesions. Hence, in patients with MS, we first obtained lesion masks in the FLAIR space using a deep learning-based tool (La Rosa et al., 2020), then we manually corrected the results and registered the lesion masks to the GRE/3DEPI native space using FSL-FLIRT (Jenkinson et al., 2012) for lesion classification using QSM.

Two raters (AC, neurologist with 4 years of experience in rating QSM contrasts and NOSS, neuroradiologist with 2 years of experience in rating QSM contrasts), performed a qualitative assessment on all derived QSM maps, blinded to the reconstruction method used. Specifically, the assessment included: (1) identification of PRLs, (2) classification of white matter lesions as QSM-hyperintense or -isointense compared to the perilesional tissue, as previously proposed (Granziera et al., 2021; Langkammer et al., 2013), and (3) identification of the CVS. Additionally, the overall image quality was rated based on the presence of (1) susceptibility artifacts, (2) motion artifacts, and (3) other artifacts. A scoring system ranging from 1 (poor quality) to 5 (excellent quality) was employed to rate the visibility and spatial definition of PRLs, the confidence in classifying WML based on QSM, the visibility and conspicuity of the CVS, and the severity of artifacts. A third expert rater (ER; biologist with 14 years of expertise in MS-related lesion assessment and segmentation) was consulted in cases where the two primary raters disagreed on lesion classification. This third rater, blinded to the reconstruction method employed, provided definitive classifications of lesions as PRLs, QSM-hyperintense or QSM-isointense, and determined the presence or absence of CVS, without assigning any qualitative scores. The derived classification consensus was used as ground truth for the calculation or sensitivity and specificity.

Before rating, the CR-based and DLR-based QSM images were de-identified for technique and patient details. A schematic representation of the processing is depicted in Supplementary Figure 2. The raters were not involved in the initial diagnosis for any of the MS cohort patients. Generally, lesions with <3 mm in size (in any plane), confluent lesions or very poorly visible lesions (due to artifacts) in FLAIR were excluded. Out of the sample size of 433 individual lesions, 12 were excluded based on the above criteria. Additionally, we followed the NAIMS criteria for CVS (Sati et al., 2016) and those described in (Rahmanzadeh et al., 2022) for QSM lesion classification.

### 2.4 Statistical analysis of images and derived scores

For the clinical aspects, all statistical analysis was done lesion-wise (on the full sample size, unless otherwise stated). Cohen’s κ was used to assess inter-rater and inter-reconstruction method scores agreement. Wilcoxon signed-rank test was used to compare the image quality intra-rater scores between the reconstruction methods as well as the inter-rater scores for each method. Sensitivity and specificity were calculated based on the following formulas: Sensitivity = TP/(TP + FN) and Specificity = TN/(TN + FP), where TP: True Positive count, TN: True Negative count, FP: False Positive count, FN: False Negative count.

Quantitative susceptibility mapping reconstructions based on the data from a healthy individual, were compared qualitatively and statistically using mean absolute error (MAE), root mean squared error (RMSE), and ROI-based evaluations of the basal ganglia and brain stem major nuclei, as done in previous QSM methodological studies (Langkammer et al., 2016b). The specific ROIs are denoted in Supplementary Figure 3. Statistical analysis was performed by DGG in Matlab 2023b (The MathWorks, Natick, MA, USA). *P* values of <0.05 were considered statistically significant.

## 3 Results

### 3.1 Visual assessment of MS and normal appearing tissue-related characteristics on DLR and CR data-based QSM in the MS cohort

**FIGURE 1:**
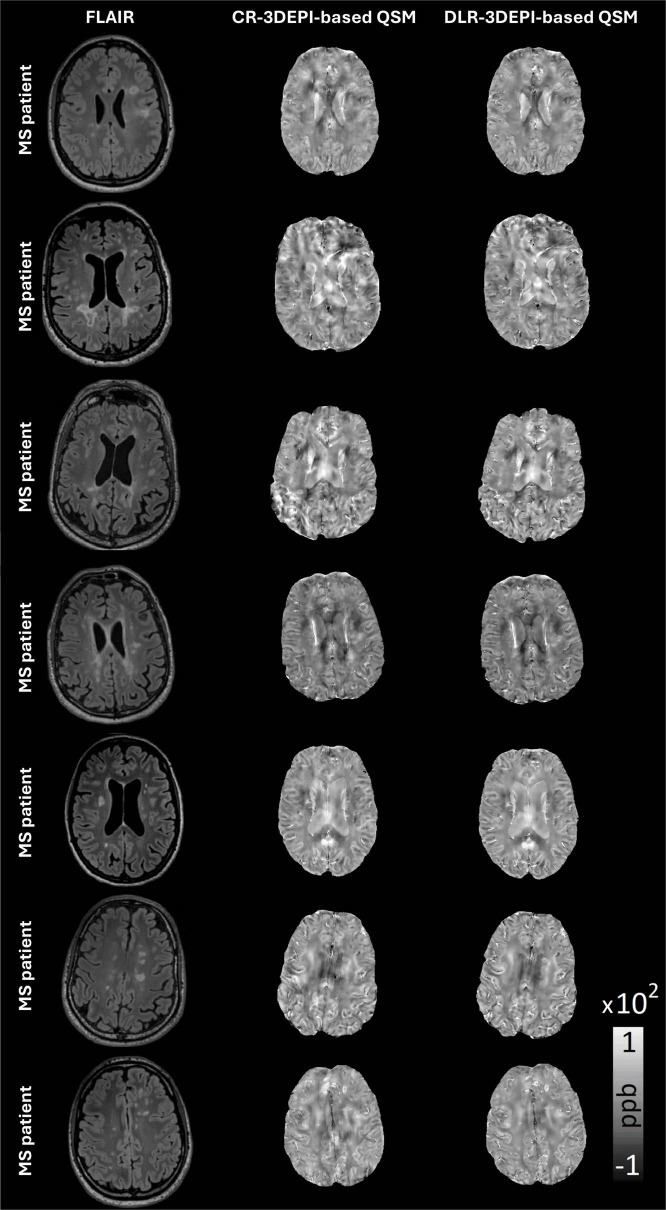
Representative slices of all MS patients FLAIR, conventionally (CR) and deep learning (DLR)-reconstructed 3DEPI data-based QSM, with a plethora of characteristic MS-related lesions. Close visual assessment reveals multiple artifacts in CR-based QSM that are cleared in DLR-based QSM offering superior depictions of the local, clinically relevant contrast in WM.

In Figure 1, representative slices of the FLAIR, CR-based and DLR-based χ-maps are presented for all patients included in the study. Characteristic MS-related lesions are abundant in careful visual assessment. Multiple artifacts present in CR-based χ-maps are alleviated in the corresponding DLR-based χ-maps, offering superior depictions of the local, clinically relevant and structural contrast in white matter lesions.

**FIGURE 2:**
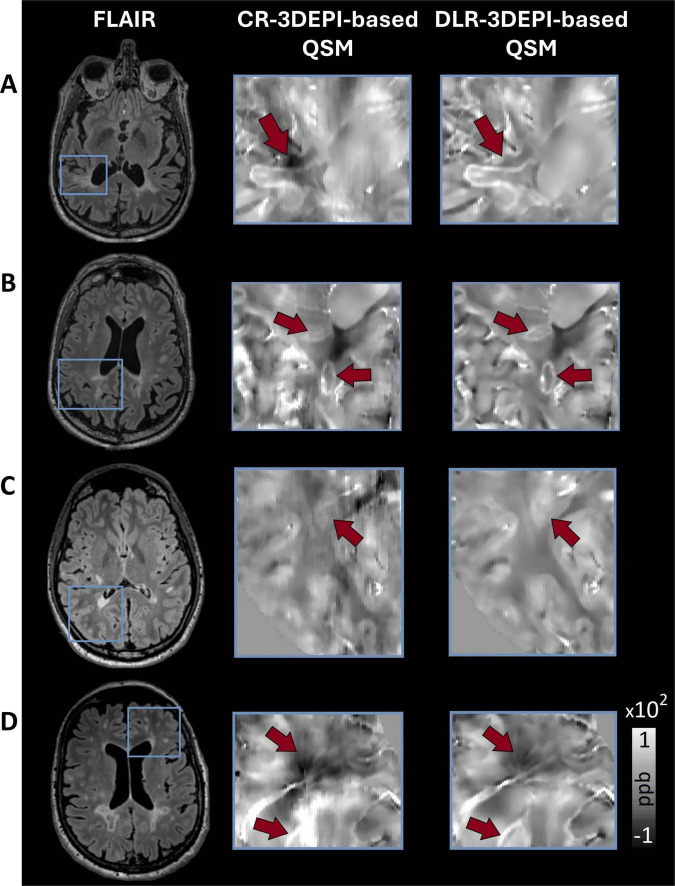
Selection of exemplary zoomed-in ROIs emphasizing on findings of clinical relevance in MS, for comparison between conventionally (CR) and deep learning (DLR)-reconstructed 3DEPI data-based QSM. **(A,B)** Due to the reduction of non-local χ effects (that cause artifacts), DLR-based QSM offers clearer depictions of the paramagnetic rims. Additionally, the structural information in the surrounding areas appears sharper and artifact-free, making the identification of the clinical landmarks easier. **(C)** A hyperintensity caused by a periventricular lesion (as seen in the FLAIR) is almost non-identifiable in the CR-based QSM, due to the susceptibility artifacts that deprecate the signal surrounding the interface of ventricles-tissue. In DLR-based QSM, the same hyperintensity is clearly identified and delineated. **(D)** The presence of vasculature and the ventricles-tissue interface in this periventricular region creates artifacts on the CR-based QSM that can be misidentified as hypointensity, whereas, in the DLR-based QSM, the artifacts are alleviated, revealing mostly isointense WM and sharper depiction of the vasculature.

In Figures 2A, B, exemplary zoomed-in ROIs from CR-based and DLR-based QSM maps obtained on 3DEPI data are presented. Sharpening and reduction of susceptibility-related artifacts led to clearer depiction of the paramagnetic rims in DLR-based QSM. Structural information in the surrounding areas also appeared sharper and artifact-free, enhancing the identification of pathological changes, within and around lesions. This is evident in Figure 2C, where a well-defined hyperintensity in DLR-based QSM caused by a periventricular lesion (as seen in the corresponding FLAIR) is almost non-identifiable in the CR-based QSM, due to the susceptibility (and interpolation) artifacts that deprecate the signal surrounding the ventricles-tissue interface. Another example for the superiority of DLR over CR is illustrated in Figure 2D: the presence of vasculature and the cerebrospinal fluid (CSF) interface with the tissue creates artifacts on the CR-based QSM that can be misidentified as hypointensities, whereas artifacts are alleviated in DLR-based QSM, revealing mostly isointense WM and sharper depiction of the vasculature.

Overall, DLR-based QSM also provided more homogeneous signal in the normal-appearing white matter (NAWM), structures and lesion border definitions. While not within the primary scope of this study, an additional quantitative assessment in different ROIs, based on the CR- and DLR-3DEPI-based QSM of patients with MS, indicated no statistically significant differences between the ROI mean values of the two pipelines, as well as lower standard deviations for DLR-3DEPI-based QSM (Supplementary Figure 4), denoting a lower percentage of outliers in the DLR-based susceptibility maps.

### 3.2 Lesion-wise comparisons of qualitative ratings based on DLR and CR data-based QSM in MS

Table 2 reports the average lesion-wise scores assigned by the two raters for each QSM biomarker of interest. The scores are averaged separately for PRLs, hyper/isointense lesions and CVS identification, as well as for cumulatively for the whole lesion sample size (433 lesions in total, 12 excluded). In all cases, qualitative scores were higher for DLR-based over CR-based QSM maps. DLR-based QSM maps provided a significant increase in raters’ confidence in classifying MS lesions based on their appearance in QSM (hyper vs. iso) (*P* < 0.001) and identifying the CVS (*P* = 0.01).

**TABLE 2:** Mean scores from expert qualitative evaluations.

Specific MS or image quality characteristics	CR-QSM	DLR-QSM	*P*-value
Paramagnetic rim lesions	2.86 ± 1.20	2.93 ± 1.18	0.78
Hyper/isointense QSM lesions	2.80 ± 0.66	2.88 ± 0.65	<0.001
Central vein sign	2.58 ± 0.62	2.61 ± 0.63	0.01
All lesions	2.70 ± 0.69	2.76 ± 0.69	<0.001
Susceptibility artifacts	2.21 ± 1.11	4.57 ± 0.35	0.01
Motion artifacts	4.10 ± 0.73	4.86 ± 0.24	0.12
Other artifacts	2.71 ± 0.91	4.57 ± 0.45	0.01
Overall quality	3.00 ± 1.19	4.67 ± 0.37	<0.001

Table indicating the scores (average ± standard deviation) of the – per lesion types – averaged scores of the two raters. The scores are presented separately for PRLs, hyper/isointense lesions and CVS identification, as well as for all MS characteristics together. CR-QSM denotes the conventionally reconstructed 3DEPI-based χ-map and the DLR-QSM denotes the deep learning- reconstructed 3DEPI-based χ-map. Similarly, scores of the raters are presented for the identification of susceptibility artifacts, motion artifacts, and other artifacts, all together comprising the overall image quality. The *P* values were obtained by statistical comparison of lesion-wise average rater scores using paired Wilcoxon signed-rank tests. Total number of included lesions: 433; excluded lesions: 12. Data represent the mean ± standard deviation of scores from both raters. Scores were compared on the lesions sample and sub-samples using the Wilcoxon signed rank tests. *P* values below 0.05 constitute the result statistically significant. CR-QSM, conventionally reconstructed 3DEPI-based QSM; DLR-QSM, deep learning-reconstructed 3DEPI-based QSM.

Table 2 also reports the average scores assigned by the two raters on the overall image quality based on the presence of susceptibility, motion, and other artifacts. DLR-based χ-maps consistently showed higher scores compared to CR-based χ-maps. There was an improvement of image quality in terms of both susceptibility artifacts (*P* = 0.01) and “Other artifacts” (*P* = 0.01). The scores assigned to image quality in terms of motion artifacts were not statistically different between the two reconstruction approaches; remarkably, the presence of motion artifacts in the original 3DEPI data was minimal. The overall image quality was significantly higher for DLR-based (4.67 ± 0.37) compared to CR-based (3.00 ± 1.19) χ-maps (*P* < 0.001). It is worth to note that the consistently lower standard deviations in the scores of DLR-based χ-maps indicate a tighter clustering of the values to the mean, hence higher agreement of the individual scores.

### 3.3 Inter-rater and inter-method agreement of the lesion-wise qualitative ratings on DLR and CR data-based QSM

Inter-rater and inter-method agreement, assessed using Cohen’s κ-scores, are presented in Table 3. For both CR and DLR-based χ-maps, inter-rater agreement analysis was similar, ranging from moderate agreement in the identification of the CVS (0.42 vs. 0.41) to excellent agreement for the identification of PRLs (0.87 vs. 1). It is of high importance to note that the inter-method (QSM based on DLR data vs. QSM based on CR data) agreement was excellent for both Rater 1 and Rater 2 in for all MS-related biomarkers (see Table 3), indicating that the rater’s perception for the individual findings does not significantly change between the two methods, but their confidence in the characterization of these findings is increased for the DLR-3DEPI-based QSM in comparison to CR-3DEPI-based QSM. For both raters, sensitivity and specificity analysis did not show any notable differences between the DLR-3DEPI-based and CR-3DEPI-based QSM (Supplementary Table 5).

**TABLE 3:** Inter-rater and inter-method agreement.

MS characteristics or Lesion type	Inter-rater		Inter-method	
	Cohen’s kappa (κ) score		Cohen’s kappa (κ) score	
	CR-QSM	DLR-QSM	Rater 1	Rater 2
Paramagnetic Rim Lesions	0.87	1	1 (37–37)	1 (38–38)
Hyper/ Isointense QSM Lesions	0.61	0.62	0.97 (98–97)	0.91 (127–124)
Central Vein Sign	0.42	0.41	0.97 (42–42)	0.86 (67–77)
All Lesions	0.63	0.63	0.98	0.92

Cohen’s kappa (κ) scores of the agreement between the raters on the classification to the different MS characteristics for each method, as well as the inter-method agreement for each rater. The scores are presented separately for PRLs, hyper/isointense lesions and CVS identification, as well as for all MS characteristics together. CR-QSM denotes the conventionally reconstructed 3DEPI-based χ-map and the DLR-QSM denotes the deep learning-reconstructed 3DEPI-based χ-map. Next to the inter-method κ-scores for rater 1 and 2, in parenthesis, the corresponding number of identified lesions for CR vs. DLR-based QSM is shown (for hyper/isointense lesions, only the number of hyperintense is shown). Inter-method κ-scores are reported as κ-score (number of CR-based QSM identified lesions – number of DLR-based QSM identified lesions). For hyper/isointense QSM lesions, only the number of hyperintense lesions is shown in the table. CR-QSM, conventionally reconstructed 3DEPI-based QSM; DLR-QSM, deep learning-reconstructed 3DEPI-based QSM.

### 3.4 Direct comparison of GRE and fast 3DEPI-based χ-maps obtained from the healthy control

**FIGURE 3:**
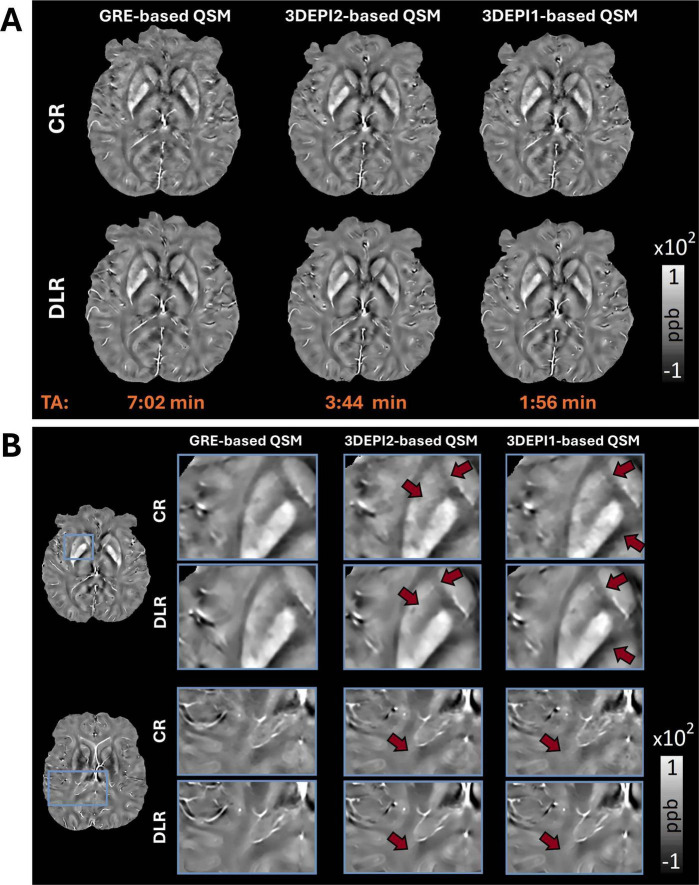
**(A)** CR-based QSM maps (1st row) derived from GRE, 3DEPI2, and 3DEPI1 (2 and 1-averages 3D-segmented-EPI) data indicate effects from artifacts and noise; especially in faster acquisition reconstructions, whereas the DLR-based QSM maps (2nd row) are showing increased SNR, reduced impact from artifacts and higher consistency. **(B)** Zoomed-in ROIs of representative slices of the control participant’s CR-based and DLR-based QSM from GRE, 3DEPI2, and 3DEPI1 (2 and 1-averages 3D-segmented-EPI data). DLR-based QSM offers clearer depictions of the expected hyperintensities in basal ganglia and brain stem nuclei (due to higher iron accumulation in these regions). In DLR-based QSM maps, most artifacts associated with abrupt χ changes such as interfaces of different structures, vasculature or movement are alleviated (see also Supplementary Figure 5, for examples, of thalamic substructures, basal ganglia, and brain stem nuclei), revealing better the characteristics of the underlying structures. Similarly, WM is more homogenous and less noisy, even in the DLR-based QSM of the fastest acquisition (single-average 3DEPI). The DLR-based QSM of all three acquisitions appears more visually similar to each other in comparison to the CR-based. CR, conventional reconstruction; DLR, deep-learning reconstruction.

In Figure 3A, a comparison of the χ-maps derived from GRE and 3DEPI2, 3DEPI1 sequences with both CR and DLR are displayed. Progressively increasing noise effects in faster acquisition reconstructions are evident for CR-based χ-maps, whereas the DLR-based χ-maps show overall higher SNR, sharper structural information. Figure 3B shows selected zoomed-in ROIs from QSM maps obtained from GRE, 3DEPI1, and 3DEPI2 with both CR and DLR. The sharper definition of structural characteristics in the DLR-based QSM is comparatively more evident in regions where high tissue iron load leads to QSM hyperintensities, e.g., basal ganglia (see also Supplementary Figure 5 for zoomed-in ROIs of thalamic and brain stem nuclei). QSM artifacts associated with abrupt changes at tissue susceptibility interfaces (GM-WM, vasculature, and sinuses) are generally reduced in DLR-based susceptibility maps. WM in DLR-based QSM appears more homogenous, even using as basis the 3DEPI1 data. Notably, the definition of the subthalamic nuclei in the DLR-based QSM is sharper and the relevant nuclei can be identified robustly, in comparison to the CR-based maps (see Supplementary Figure 6).

Statistical assessment of the QSM estimations indicated: (i) RMSE for 3DEPI1 vs. GRE and 3DEPI2 vs. GRE was slightly lower for DLR-based QSM (20.4%, 19.4%) vs. CR-based QSM (21.5%, 21%), (ii) MAE indicating a similar trend of reduction, to 4.16 ppb, 4.03 ppb, from 4.98 ppb, 4.41 ppb of CR-based. The assessment of the major brainstem/basal ganglia nuclei showed a reduction of the mean differences between GRE and 3DEPI DLR-based QSM, to 1.61 ppb, from 2.48 ppb for CR-based. The standard deviations of GRE, 3DEPI1, and 3DEPI2 DLR-based QSM in the different ROIs were reduced in comparison to CR-based (see Supplementary Table 4), indicating a potentially lower percentage of outliers present in the DLR-based χ maps.

## 4 Discussion

In this pilot study, the combination of fast 3DEPI MRI acquisitions and a novel, k-space operating AI-based denoising, reconstruction and super-resolution (“DLR”) pipeline was evaluated for obtaining QSM maps for the characterization of individual lesions in MS. We showed through a thorough, lesion-wise assessment that DLR-3DEPI-based QSM provided non-inferior or superior quality in supporting the identification of susceptibility imaging biomarkers of great importance in characterizing pathological changes in patients with MS, in comparison to CR-3DEPI-based QSM. These improvements were independent of the rater’s perception of the individual clinical findings. Additionally, we included in this study a first quantitative/qualitative demonstration of the DLR efficiency in enhancing the quality of very fast (fourfold decrease in acquisition time compared to GRE) 3DEPI acquisitions – that are generally more susceptible to noise and artifacts – resulting in QSM of comparable quality to the one obtained based on GRE data with matching parameters and resolution.

In recent years, numerous AI-based methods have been proposed and shown to improve image quality across various MRI contrasts (Pfeuffer et al., 2024; Gong et al., 2018; Bahrami et al., 2019; Do et al., 2020; Rudie et al., 2022; Kiryu et al., 2023). However, it is crucial to separately evaluate the application of these AI methodologies for each case of pathological characterization to assess their potential benefits and to ensure that they maintain clinical utility. This can only be achieved through expert evaluations. In our study, the DLR-3DEPI-based χ-maps were consistently rated higher than CR-3DEPI-based χ-maps for individual MS pathological findings and in terms of artifacts and overall image quality. This improvement is likely due to the denoising and super-resolution capabilities of the DLR pipeline. At the same time, inter-method agreement was excellent for both raters, indicating that the DLR-3DEPI-derived χ-maps do not alter the rater’s perception of the individual findings but increase their confidence in the characterizing them, in comparison to CR-3DEPI-based QSM assessments.

It is worth noting that inter-rater Cohen’s kappa was lower for CVS compared to other MS-related biomarkers, such as PRLs. While QSM has been proven to be an optimal method for the identification of PRLs (Bagnato et al., 2024), it is still under evaluation for its application in detecting the CVS (Sati et al., 2016; Li et al., 2022). Given that blood vessels differ in their susceptibility properties depending on the state of hemoglobin oxygenation (oxygen-saturated hemoglobin is weakly diamagnetic, while deoxyhemoglobin is paramagnetic) (Eldeniz et al., 2021), the identification of CVS becomes more ambiguous depending on the QSM signal of the lesion core (hyper-, iso/hypo-intense). This variability likely contributes to the reduced inter-rater agreement, alongside other factors that may obscure smaller structures in QSM, such as regularization effects. Previous studies assessing the use of QSM for CVS identification have reported lower percentages of lesions observed with CVS on QSM (Zhang et al., 2019) compared to earlier studies employing other imaging techniques (Maggi et al., 2018).

While this study primarily focuses on the qualitative assessment of the two raters, we also conducted a quantitative investigation of the comparability between QSM maps obtained based on conventional GRE sequences and two 3DEPI protocols (with progressively faster acquisition times) for both DLR and CR. The results clearly indicated that the consistency, sharpness and SNR of the DLR χ-maps is increased while the effect of artifacts is decreased, despite the lower SNR in the faster-acquired 3DEPI measurements. Given the paramount importance of time in clinical settings, the potential of a fourfold reduction in examination time holds significant potential for advancing the broader clinical adoption of QSM, with reduced costs and efficient utilization of scanner resources, not only in MS, but also to a broader spectrum of pathologies involving potential susceptibility imaging-sensitive alterations (Shibukawa et al., 2024; Gkotsoulias et al., 2025; Langkammer et al., 2016a; Cogswell et al., 2021). Despite the obvious advantages of rapid DLR-3DEPI-based QSM-shown here on a healthy brain, additional studies are needed to ensure the clinical utility of specifically these fast protocols in MS or other pathologies. It is worth noting that the QSM maps obtained between the two parts of the study are not comparable: (i) the clinical scans were acquired with a 0.67 mm isotropic resolution, whereas the scans of the control subject were obtained with a 0.7 mm × 0.7 mm × 2 mm resolution, providing higher SNR per voxel due to the increased voxel volume. While this setup is optimized for speed and clinical feasibility, it is less suitable for extracting microstructural information. (ii) In the MRI scans of MS patients, motion susceptibility is higher due to the extended scanning duration (more than one measurement is acquired), while in general, the shimming is automatically set. In contrast, the single control subject MRI scans were performed solely for the purposes of this analysis, with shorter scan times and careful attention to parameters like shimming and head positioning, aiming to compare faster and slower protocols based QSM.

Our study comes with a number of limitations. First, the MS cohort in this pilot study was limited in size, comprising seven patients. However, the nature of the presented assessments is lesion-based and not patient-based (except when it comes to overall QSM image quality metrics). Hence, the high lesion load in all subjects ensured a sample size of 433 lesions and can provide reassurance regarding the generalizability of the findings. Second, all patients were scanned with a single 3T MRI system; Future evaluations of the method would benefit from the inclusion of larger patient cohorts and different clinical MRI scanner strengths (above and below 3T).

The recent QSM Consensus (QSM Consensus Organization Committee et al., 2024) guidelines suggest that using isotropic voxels and multi-echo schemes in the QSM-related acquisitions is preferable. While in terms of physical underpinnings-modeling of QSM this is indeed the optimal case (Schweser et al., 2016; Deistung et al., 2017), we made the selection of the parameters always keeping in mind the limitations of the specific 3DEPI implementation and the strong time constraints posed—especially for newly introduced methods— in the clinical environment: For the clinical evaluations, the native resolution of the protocols used is isotropic while in the secondary part of the study that introduces the faster protocols, the native resolution was 0.7 mm × 0.7 mm × 2 mm (both axially interpolated post-acquisition, by a factor of 2). GRE was constrained in single echo, for fair comparison to the 3DEPI-derived QSM. Methodologically, while the TGV is a very prominent and recently proposed method, it would be interesting in future studies to include more methodologies for QSM estimation. It is worth noting that while the number of raters (2) may seem limited, they both had specific experience in conducting QSM assessments in large, multi-center cohorts of MS patients – a third experienced rater was employed to ensure that classification consensus in the cases of disagreement. Last, it is important to mention that the susceptibility values within a WM lesion and their difference to the surrounding tissue are influenced by the location and local microstructure of the brain tissue, including myelin density, iron deposition and orientation characteristics of WM myelinated fibers and other factors (Deistung et al., 2017; Gkotsoulias et al., 2024; Li et al., 2016; Li et al., 2011). For that reason, we proceeded with a qualitative, analysis as it is done in clinical practice; in this context, the improvement observed in the ratings reflects rather an improved image quality and contrast to surroundings of DLR-derived QSM.

## 5 Conclusion

According to expert ratings, the proposed vendor-specific DLR-3DEPI provides basis-data that lead to substantially increased QSM quality and clinical utility for the identification of MS-specific biomarkers, without changing the expert’s perception of the individual findings compared to the CR-3DEPI-based QSM. DLR seems to be also efficient in increasing the quality and reducing the artifacts of rapid 3DEPI protocols with fourfold-reduced acquisition time compared to the state-of-the-art GRE, leading to QSM visually and quantitatively comparable to GRE-based. These findings hold significant promise for advancing the broader implementation of DLR-3DEPI-based QSM in the management of patients with MS.

## Data Availability

The datasets presented in this article are not readily available because of patient confidentiality agreements. The raw imaging data can be made available upon request from the corresponding author, upon considerations. Requests to access the datasets should be directed to dimitrios.gkotsoulias@unibas.ch.

## References

[B1] Absinta M. Sati P. Masuzzo F. Nair G. Sethi V. Kolb H. (2019). Association of chronic active multiple sclerosis lesions with disability in vivo. *JAMA Neurol.* 76 1474–1483. 10.1001/jamaneurol.2019.2399 31403674 PMC6692692

[B2] Afat S. Wessling D. Afat C. Nickel D. Arberet S. Herrmann J. (2022). Analysis of a deep learning-based superresolution algorithm tailored to partial fourier gradient echo sequences of the abdomen at 1.5 T: Reduction of breath-hold time and improvement of image quality. *Invest. Radiol.* 57 157–162. 10.1097/RLI.0000000000000825 34510101

[B3] Almansour H. Gassenmaier S. Nickel D. Kannengiesser S. Afat S. Weiss J. (2021). Deep learning-based superresolution reconstruction for upper abdominal magnetic resonance imaging: An analysis of image quality, diagnostic confidence, and lesion conspicuity. *Invest. Radiol.* 56 509–516. 10.1097/RLI.0000000000000769 33625063

[B4] Bagnato F. Sati P. Hemond C. Elliott C. Gauthier S. Harrison D. (2024). Imaging chronic active lesions in multiple sclerosis: A consensus statement. *Brain* 147 2913–2933. 10.1093/brain/awae013 38226694 PMC11370808

[B5] Bahrami K. Shi F. Zong X. Shin H. An H. Shen D. (2019). Reconstruction of 7T-Like Images From 3T MRI. *IEEE Trans. Med. Imaging* 35 2085–2097. 10.1109/TMI.2016.2549918 27046894 PMC5147737

[B6] Behl N. (2021). *Deep resolve – mobilizing the power of networks (White paper) MAGNETOM flash (78):1/2021.* Erlangen: Siemens Healthineers.

[B7] Cagol A. Benkert P. Melie-Garcia L. Schaedelin S. Leber S. Tsagkas C. (2024a). Association of spinal cord atrophy and brain paramagnetic rim lesions with progression independent of relapse activity in people with MS. *Neurology* 102:e207768. 10.1212/WNL.0000000000207768 38165377 PMC10834139

[B8] Cagol A. Cortese R. Barakovic M. Schaedelin S. Ruberte E. Absinta M. (2024b). Diagnostic performance of cortical lesions and the central vein sign in multiple sclerosis. *JAMA Neurol.* 81 143–153. 10.1001/jamaneurol.2023.4737 38079177 PMC10714285

[B9] Castellaro M. Tamanti A. Pisani A. Pizzini F. Crescenzo F. Calabrese M. (2020). The use of the central vein sign in the diagnosis of multiple sclerosis: A systematic review and meta-analysis. *Diagnostics (Basel)* 10:1025. 10.3390/diagnostics10121025 33260401 PMC7760678

[B10] Chaika M. Afat S. Wessling D. Afat C. Nickel D. Kannengiesser S. (2023). Deep learning-based super-resolution gradient echo imaging of the pancreas: Improvement of image quality and reduction of acquisition time. *Diagn. Interv. Imaging* 104 53–59. 10.1016/j.diii.2022.06.006 35843839

[B11] Cogswell P. Wiste H. Senjem M. Gunter J. Weigand S. Schwarz C. (2021). Associations of quantitative susceptibility mapping with Alzheimer’s disease clinical and imaging markers. *Neuroimage* 224:117433. 10.1016/j.neuroimage.2020.117433 33035667 PMC7860631

[B12] Deistung A. Schweser F. Reichenbach J. (2017). Overview of quantitative susceptibility mapping. *NMR Biomed.* 30:69. 10.1002/nbm.3569 27434134

[B13] Dimov A. Gillen K. Nguyen T. Kang J. Sharma R. Pitt D. (2022). Magnetic susceptibility source separation solely from gradient echo data: Histological validation. *Tomography* 8 1544–1551. 10.3390/tomography8030127 35736875 PMC9228115

[B14] Do W. Seo S. Han Y. Ye J. Choi S. Park S. (2020). Reconstruction of multicontrast MR images through deep learning. *Med. Phys.* 47 983–997. 10.1002/mp.14006 31889314

[B15] Eldeniz C. Binkley M. Fields M. Guilliams K. Ragan D. Chen Y. (2021). Bulk volume susceptibility difference between deoxyhemoglobin and oxyhemoglobin for HbA and HbS: A comparative study. *Magn. Reson. Med.* 85 3383–3393. 10.1002/mrm.28668 33475200 PMC7945010

[B16] Frahm J. Haase A. Matthaei D. (1986). Rapid NMR imaging of dynamic processes using the FLASH technique. *Magn. Reson. Med.* 3 321–327. 10.1002/mrm.1910030217 3713496

[B17] Gillen K. Mubarak M. Park C. Ponath G. Zhang S. Dimov A. (2021). QSM is an imaging biomarker for chronic glial activation in multiple sclerosis lesions. *Ann. Clin. Transl. Neurol.* 8 877–886. 10.1002/acn3.51338 33704933 PMC8045922

[B18] Gkotsoulias D. Jäger C. Müller R. Gräßle T. Olofsson K. Møller T. (2024). Chaos and COSMOS-Considerations on QSM methods with multiple and single orientations and effects from local anisotropy. *Magn. Reson. Imaging* 110 104–111. 10.1016/j.mri.2024.04.020 38631534

[B19] Gkotsoulias D. Rullmann M. Schmitt S. Bujanow A. Zientek F. Messerschmidt K. (2025). Abnormalities of iron homeostasis and the dopaminergic system in Tourette syndrome revealed by 7T MRI and PET. *Brain Commun.* 7:fcaf104. 10.1093/braincomms/fcaf104 40177529 PMC11961303

[B20] Gong E. Pauly J. Wintermark M. Zaharchuk G. (2018). Deep learning enables reduced gadolinium dose for contrast-enhanced brain MRI. *J. Magn. Reson. Imaging* 48 330–340. 10.1002/jmri.25970 29437269

[B21] Granziera C. Wuerfel J. Barkhof F. Calabrese M. De Stefano N. Enzinger C. (2021). Quantitative magnetic resonance imaging towards clinical application in multiple sclerosis. *Brain* 144 1296–1311. 10.1093/brain/awab029 33970206 PMC8219362

[B22] Hammernik K. Klatzer T. Kobler E. Recht M. Sodickson D. Pock T. (2018). Learning a variational network for reconstruction of accelerated MRI data. *Magn. Reson. Med.* 79 3055–3071. 10.1002/mrm.26977 29115689 PMC5902683

[B23] Hammernik K. Knoll F. Rueckert D. (2019). *Deep learning for parallel MRI reconstruction. MAGNETOM Flash (75):4/2019.* London: Imperial College London, 10–15.

[B24] Huang W. Sweeney E. Kaunzner U. Wang Y. Gauthier S. Nguyen T. (2022). Quantitative susceptibility mapping versus phase imaging to identify multiple sclerosis iron rim lesions with demyelination. *J. Neuroimaging* 32 667–675. 10.1111/jon.12987 35262241 PMC9308704

[B25] Jenkinson M. Beckmann C. Behrens T. Woolrich M. Smith S. M. (2012). FSL. *Neuroimage* 62 782–790. 10.1016/j.neuroimage.2011.09.015 21979382

[B26] Kaunzner U. Kang Y. Zhang S. Morris E. Yao Y. Pandya S. (2019). Quantitative susceptibility mapping identifies inflammation in a subset of chronic multiple sclerosis lesions. *Brain* 142 133–145. 10.1093/brain/awy296 30561514 PMC6308309

[B27] Kingma D. P. Jimmy B. (2014). Adam: A method for stochastic optimization. *arXiv* [Preprint]. arXiv:1412.6980.

[B28] Kiryu S. Akai H. Yasaka K. Tajima T. Kunimatsu A. Yoshioka N. (2023). Clinical impact of deep learning reconstruction in MRI. *Radiographics* 43:e220133. 10.1148/rg.220133 37200221

[B29] La Rosa F. Abdulkadir A. Fartaria M. Rahmanzadeh R. Lu P. Galbusera R. (2020). Multiple sclerosis cortical and WM lesion segmentation at 3T MRI: A deep learning method based on FLAIR and MP2RAGE. *Neuroimage Clin.* 27:102335. 10.1016/j.nicl.2020.102335 32663798 PMC7358270

[B30] Langkammer C. Bredies K. Poser B. Barth M. Reishofer G. Fan A. (2015). Fast quantitative susceptibility mapping using 3D EPI and total generalized variation. *Neuroimage* 111 622–630. 10.1016/j.neuroimage.2015.02.041 25731991

[B31] Langkammer C. Liu T. Khalil M. Enzinger C. Jehna M. Fuchs S. (2013). Quantitative susceptibility mapping in multiple sclerosis. *Radiology* 267 551–559. 10.1148/radiol.12120707 23315661 PMC3632806

[B32] Langkammer C. Schweser F. Shmueli K. Kames C. Li X. Guo L. (2016b). Quantitative susceptibility mapping: Report from the 2016 reconstruction challenge. *Magn. Reson. Med.* 79 1661–1673. 10.1002/mrm.26830 28762243 PMC5777305

[B33] Langkammer C. Pirpamer L. Seiler S. Deistung A. Schweser F. Franthal S. (2016a). Quantitative susceptibility mapping in Parkinson’s disease. *PLoS One* 11:e0162460. 10.1371/journal.pone.0162460 27598250 PMC5012676

[B34] Li J. Huang W. Luo X. Wen Y. Cho J. Kovanlikaya I. (2022). The central vein sign in multiple sclerosis lesions: Susceptibility relaxation optimization from a routine MRI multiecho gradient echo sequence. *J. Neuroimaging* 32 48–56. 10.1111/jon.12938 34664747

[B35] Li W. Wu B. Liu C. (2011). Quantitative susceptibility mapping of human brain reflects spatial variation in tissue composition. *Neuroimage* 55 1645–1656. 10.1016/j.neuroimage.2010.11.088 21224002 PMC3062654

[B36] Li X. Harrison D. Liu H. Jones C. Oh J. Calabresi P. (2016). Magnetic susceptibility contrast variations in multiple sclerosis lesions. *J. Magn. Reson. Imaging* 43 463–473. 10.1002/jmri.24976 26073973 PMC4678033

[B37] Lopez Schmidt I. Haag N. Shahzadi I. Frohwein L. Schneider C. Niehoff J. (2023). Diagnostic image quality of a low-field (0.55T) knee MRI protocol using deep learning image reconstruction compared with a standard (1.5T) knee MRI protocol. *J. Clin. Med.* 12:1916. 10.3390/jcm12051916 36902704 PMC10003576

[B38] Maggi P. Absinta M. Grammatico M. Vuolo L. Emmi G. Carlucci G. (2018). Central vein sign differentiates Multiple Sclerosis from central nervous system inflammatory vasculopathies. *Ann. Neurol.* 83 283–294. 10.1002/ana.25146 29328521 PMC5901412

[B39] Martire M. Moiola L. Rocca M. Filippi M. Absinta M. (2022). What is the potential of paramagnetic rim lesions as diagnostic indicators in multiple sclerosis? *Expert Rev. Neurother.* 22 829–837. 10.1080/14737175.2022.2143265 36342396

[B40] Möller H. Bossoni L. Connor J. Crichton R. Does M. Ward R. (2019). Iron, myelin, and the brain: Neuroimaging meets neurobiology. *Trends Neurosci.* 42 384–401. 10.1016/j.tins.2019.03.009 31047721

[B41] Paszke A. Gross S. Massa F. Lerer A. Bradbury J. Chanan G. (2019). “PyTorch: An imperative style, high-performance deep learning library,” in *Proceedings of the 33rd conference on neural information processing systems*, (Vancouver, BC).

[B42] Pfeuffer J. Eckstein K. Stewart A. (2024). *Advances in susceptibility-weighted imaging (SWI) and quantitative susceptibility mapping (QSM), MAGNETOM Flash (87):2/2024.* Brisbane, QL: University of Queensland.

[B43] Preziosa P. Rocca M. Filippi M. (2021). Central vein sign and iron rim in multiple sclerosis: Ready for clinical use? *Curr. Opin. Neurol.* 34 505–513. 10.1097/WCO.0000000000000946 33928930

[B44] QSM Consensus Organization Committee, Bilgic B. Costagli M. Chan K. S. Duyn J. Langkammer C. (2024). Recommended implementation of quantitative susceptibility mapping for clinical research in the brain: A consensus of the ISMRM electro-magnetic tissue properties study group. *Magn. Reson. Med.* 91 1834–1862. 10.1002/mrm.30006 38247051 PMC10950544

[B45] Rahmanzadeh R. Galbusera R. Lu P. Bahn E. Weigel M. Barakovic M. (2022). A new advanced MRI biomarker for remyelinated lesions in multiple sclerosis. *Ann. Neurol.* 92 486–502. 10.1002/ana.26441 35713309 PMC9527017

[B46] Rudie J. Gleason T. Barkovich M. Wilson D. Shankaranarayanan A. Zhang T. (2022). Clinical assessment of deep learning-based super-resolution for 3D volumetric brain MRI. *Radiol. Artif. Intell.* 4:e210059. 10.1148/ryai.210059 35391765 PMC8980882

[B47] Sati P. Oh J. Constable R. Evangelou N. Guttmann C. Henry R. (2016). The central vein sign and its clinical evaluation for the diagnosis of multiple sclerosis: A consensus statement from the North American imaging in multiple sclerosis cooperative. *Nat. Rev. Neurol.* 12 714–722. 10.1038/nrneurol.2016.166 27834394

[B48] Sati P. Thomasson D. Li N. Pham D. Biassou N. Reich D. (2014). Rapid, high-resolution, whole-brain, susceptibility-based MRI of multiple sclerosis. *Mult. Scler.* 20 1464–1470. 10.1177/1352458514525868 24639479 PMC4167170

[B49] Schweser F. Deistung A. Reichenbach J. (2016). Foundations of MRI phase imaging and processing for quantitative susceptibility mapping (QSM). *Z. Med. Phys.* 26 6–34. 10.1016/j.zemedi.2015.10.002 26702760

[B50] Shibukawa S. Kan H. Honda S. Wada M. Tarumi R. Tsugawa S. (2024). Alterations in subcortical magnetic susceptibility and disease-specific relationship with brain volume in major depressive disorder and schizophrenia. *Transl. Psychiatry* 14:164. 10.1038/s41398-024-02862-7 38531856 PMC10965930

[B51] Stewart A. Robinson S. O’Brien K. Jin J. Widhalm G. Hangel G. (2022). QSMxT: Robust masking and artifact reduction for quantitative susceptibility mapping. *Magn. Reson. Med.* 87 1289–1300. 10.1002/mrm.29048 34687073 PMC7612305

[B52] Tourell M. Jin J. Bachrata B. Stewart A. Ropele S. Enzinger C. (2024). Three-dimensional EPI with shot-selective CAIPIRIHANA for rapid high-resolution quantitative susceptibility mapping at 3 T. *Magn. Reson. Med.* 92 997–1010. 10.1002/mrm.30101 38778631

[B53] Wang Y. Spincemaille P. Liu Z. Dimov A. Deh K. Li J. (2017). Clinical quantitative susceptibility mapping (QSM): Biometal imaging and its emerging roles in patient care. *J. Magn. Reson. Imaging* 46 951–971. 10.1002/jmri.25693 28295954 PMC5592126

[B54] Zhang S. Nguyen T. Hurtado Rúa S. Kaunzner U. Pandya S. Kovanlikaya I. (2019). Quantitative susceptibility mapping of time-dependent susceptibility changes in multiple sclerosis lesions. *AJNR Am. J. Neuroradiol.* 40 987–993. 10.3174/ajnr.A6071 31097429 PMC6565472

