# Supplementary material for: A pilot study assessing the clinical utility of deep learning-reconstructed 3D-echo-planar-imaging-based quantitative susceptibility mapping in multiple sclerosis
Source: Front Neurosci. 2025 Jul 16;19:1544376. doi: 10.3389/fnins.2025.1544376 (PMC12307354; doi:10.3389/fnins.2025.1544376)
Supplement: Supplementary file 1 [file Data_Sheet_1.pdf]

SUPPLEMENTARY MATERIAL

A pilot study for assessing the clinical utility of deep learning-reconstructed 3D-Echo-Planar-Imaging-based Quantitative Susceptibility Mapping in Multiple Sclerosis

Dimitrios G. Gkotsoulias, PhD[1,2,3], Matthias Weigel, PhD[1,2,3,4], Alessandro Cagol, MD[1,2,3,5], Nina de Oliveira Soares Siebenborn, MD[1,2,3], Esther Ruberte, PhD[1,2,3], Josef Pfeuffer, PhD[6], Cristina Granziera,MD, PhD[1,2,3]

[1] Department of Neurology & Multiple Sclerosis Centre, University Hospital and University of Basel, Basel, Switzerland

[2] Research Center for Clinical Neuroimmunology and Neuroscience Basel, University Hospital and University of Basel, Switzerland

[3] Translational Imaging in Neurology Basel, Department of Biomedical Engineering, Faculty of Medicine, University Hospital Basel and University of Basel, Basel, Switzerland

[4] Division of Radiological Physics, Department of Radiology, University Hospital Basel, Basel, Switzerland

[5] Department of Health Sciences, University of Genova, 16132 Genova, Italy

[6] Application Development, Siemens Healthineers AG, Erlangen, Germany

---

Supplementary Material SF1. Schematic of the pipeline for the processing of the healthy control data.

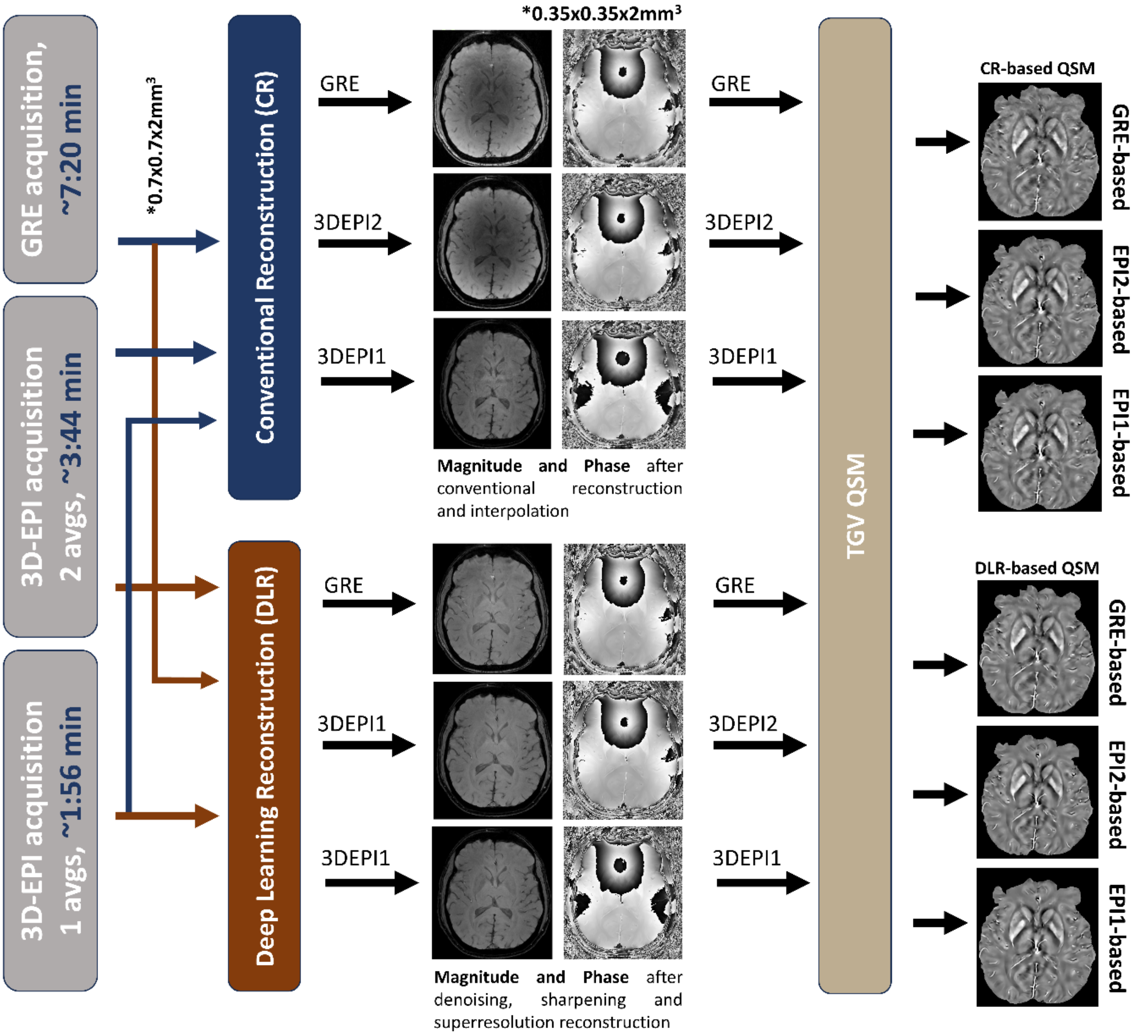

Supplementary Material SF2. Schematic of the pipeline for the processing of the MS cohort.

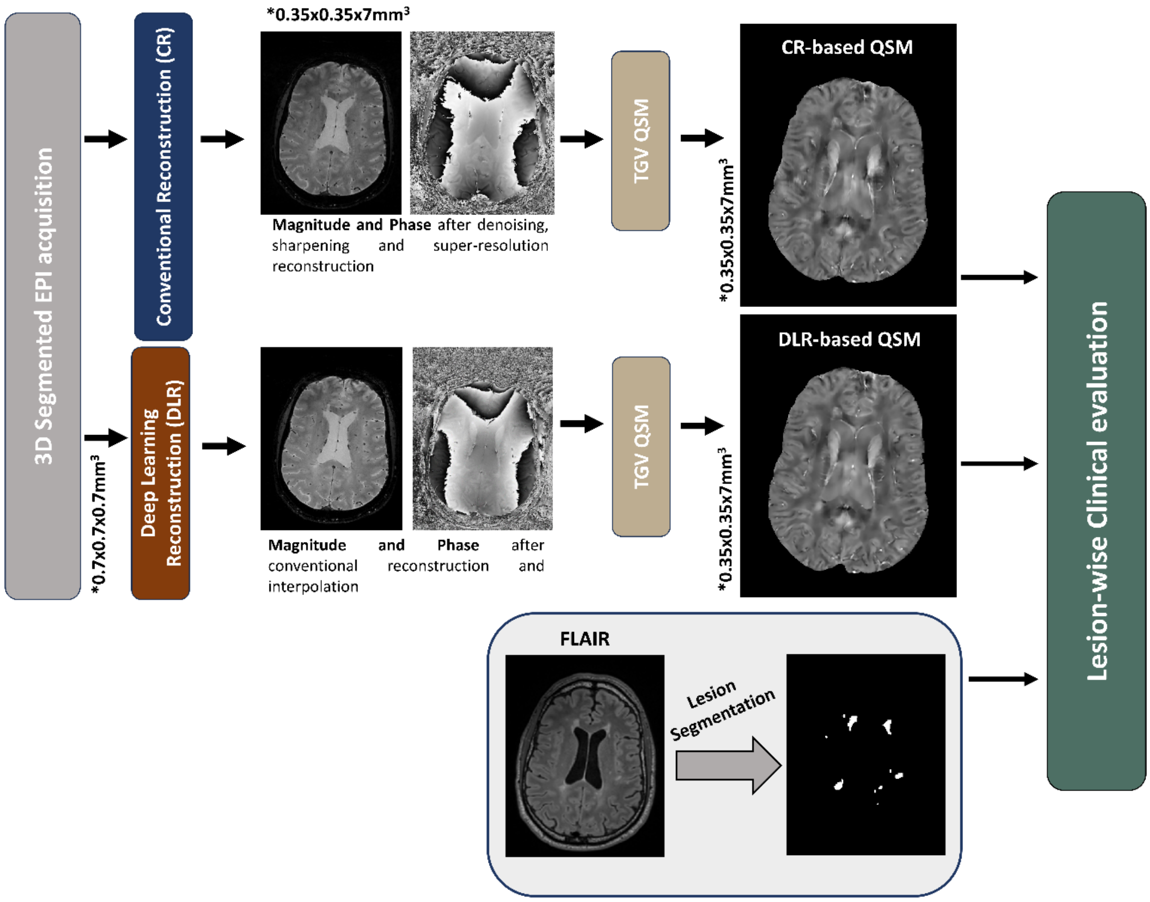

Supplementary Material SF3. Selected slices of GRE magnitude image, with depiction of the basal ganglia and brain stem major nuclei segmentation as used in parts of the healthy control QSM quantitative evaluations. **Purple**: Putamen, **Green**: Pallidum, **Blue**: Caudate, **Red**: Red Nucleus, Yellow: Substantia Nigra.

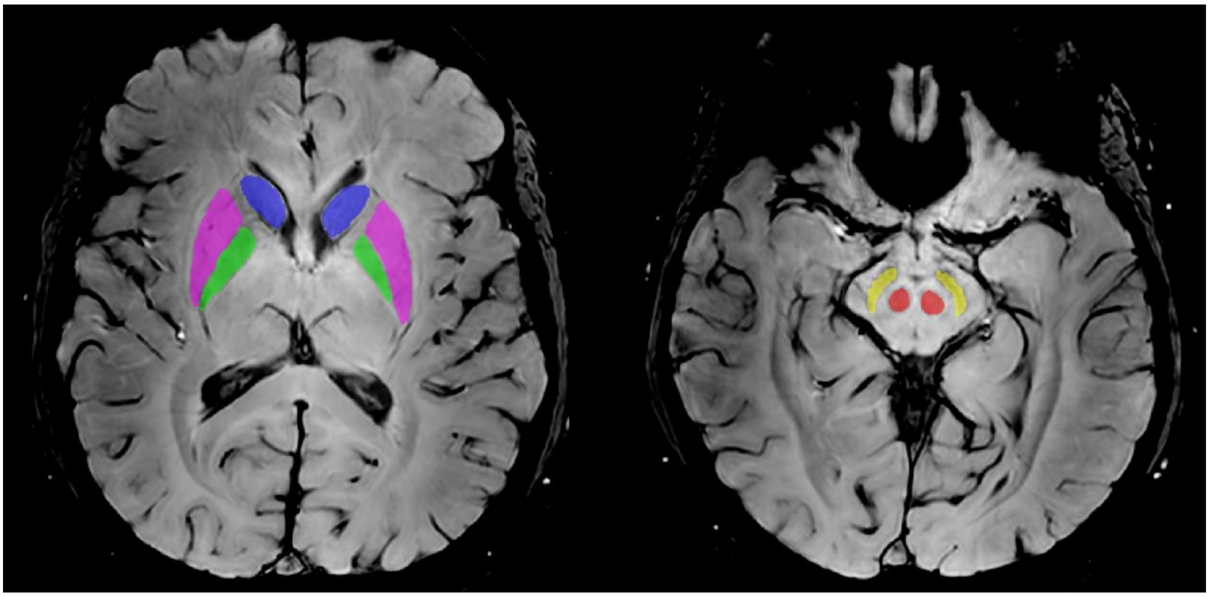

Supplementary Material SF4. Violin plots of the mean values **(A)** and analytically, the Mean ± SD values **(B)** of CR-3DEPI-based and DLR-3DEPI-based QSM in CSF, WM lesions (all), NAWM, NADGM, Thalamus and PRLs for all patients involved in the clinical evaluations of the study. Notably, the SD remains lower for DLR-3DEPI-based QSM in all regions, in comparison to CR-3DEPI-based QSM. The differences between the means of DLR-3DEPI-based and CR-3DEPI-based QSM were not statistically significant in any of the ROIs. Patient #4 had no PRLs identified.

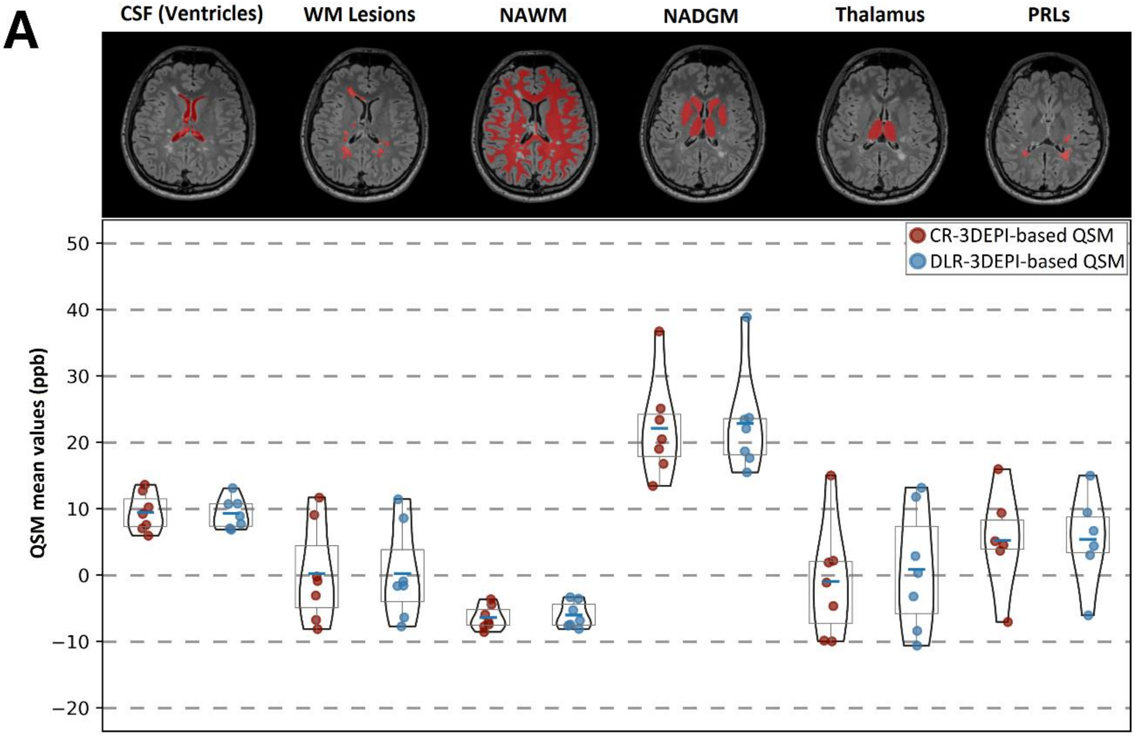

B

		CSF (Ventricles)	WM Lesions	NAWM	NADGM	Thalamus	PRLs
CR-3DEPI-based QSM	Patient 1	5.95 ± 44.64	9.07 ± 34.32	-7.43 ± 29.94	36.72 ± 81.62	15.01 ± 59.37	9.35 ± 35.34
	Patient 2	12.72 ± 35.14	11.68 ± 23.41	-5.97 ± 25.70	19.00 ± 47.72	1.89 ± 36.69	15.96 ± 21.10
	Patient 3	13.61 ± 38.98	-6.73 ± 33.32	-4.41 ± 34.70	13.42 ± 60.85	-9.86 ± 43.35	-7.04 ± 34.21
	Patient 4	9.22 ± 34.88	-8.12 ± 37.71	-3.62 ± 30.31	23.39 ± 67.35	-1.12 ± 43.00	-
	Patient 5	10.23 ± 28.62	-0.88 ± 33.95	-7.71 ± 24.86	16.76 ± 51.90	-9.96 ± 29.06	3.67 ± 35.02
	Patient 6	7.02 ± 35.87	-3.07 ± 35.68	-7.05 ± 29.42	25.09 ± 65.12	-4.66 ± 38.84	4.51 ± 36.54
	Patient 7	7.55 ± 48.18	-0.20 31.66	-8.56 ± 26.83	20.47 ± 53.20	2.17 ± 59.73	5.12 ± 31.55
DLR-3DEPI-based QSM	Patient 1	7.02 ± 43.02	8.58 ± 33.07	-7.44 ± 28.79	38.86 ± 79.29	13.17 ± 51.59	9.43 ± 34.33
	Patient 2	13.09 ± 34.87	11.43 ± 23.42	-5.27 ± 24.39	18.64 ± 47.55	2.88 ± 35.84	14.98 ± 21.43
	Patient 3	10.69 ± 37.77	-6.36 ± 29.87	-3.32 ± 30.25	15.47 ± 57.53	-8.38 ± 38.19	-6.05 ± 31.02
	Patient 4	7.70 ± 33.85	-7.71 ± 29.90	-3.54 ± 27.55	23.44 ± 66.42	0.32 ± 42.65	-
	Patient 5	10.77 ± 28.97	-1.55 ± 33.07	-7.59 ± 24.17	17.65 ± 52.26	-10.61 ± 29.42	3.04 ± 34.12
	Patient 6	8.90 ± 32.78	-1.63 ± 33.62	-6.84 ± 27.85	23.69 ± 62.41	-3.21 ± 37.78	6.67 ± 34.21
	Patient 7	6.84 ± 44.51	-0.93 ± 26.82	-8.10 ± 23.50	22.08 ± 48.36	11.79 ± 43.64	4.39 ± 25.06

All numbers are in ppb, ROI mean ± standard deviation.
CSF: Celebrospinal fluid; **NAWM**: Normal Appearing White Matter; **NADGM**: Normal Appearing Deep Gray Matter (here: Thalamus, Putamen, Caudate, Pallidum combined); **PRLs**: Paramagnetic Rim Lesions.

Supplementary Material SF5. Zoomed-in brainstem nuclei and thalamic regions on the QSM maps derived based on DLR and CR-GRE, 3DEPI2 and 3DEPI1.

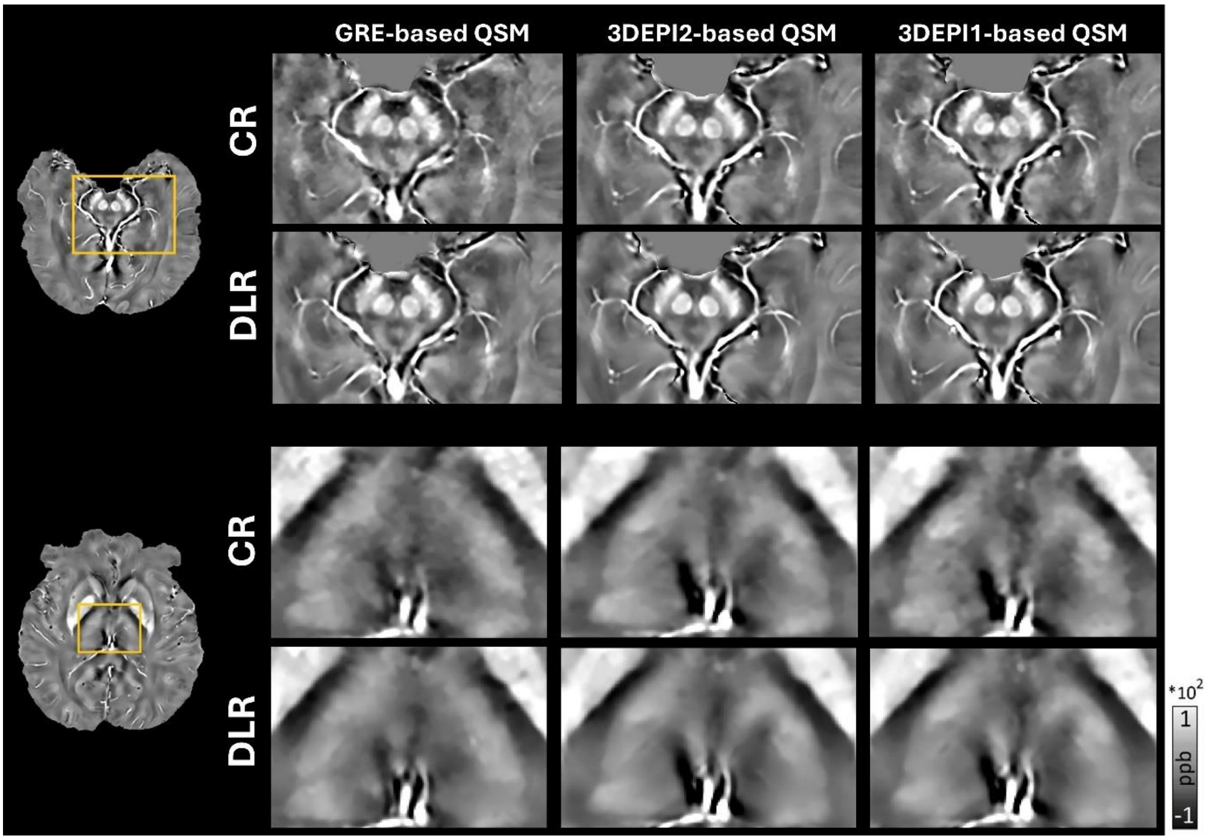

Supplementary Material SF6. In DLR 3DEPI data-based QSM estimations, subthalamic nuclei are more pronounced, smoother in intensities and with more definite borders in comparison to the CR-based maps. A manual segmentation on a single-slice zoomed-in Thalamus DLR 3DEPI1-based QSM image (by DGG, based only on the QSM intensities) follows the Morel's atlas thalamic parcellation[1] quite well. Achieving a similar segmentation on a CR 3DEPI1-BASED QSM image would not be possible due to the residual artifacts.

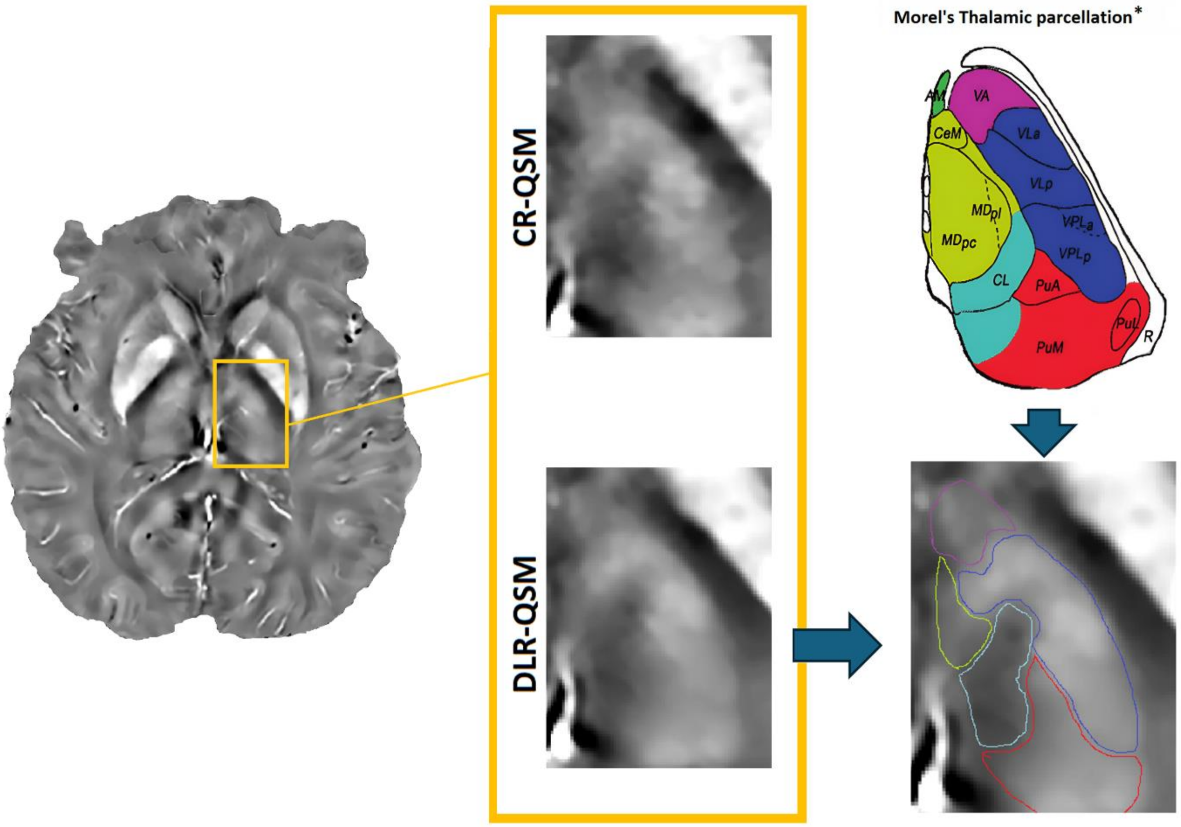

[1] Morel A, Magnin M, Jeanmonod D. Multiarchitectonic and stereotactic atlas of the human thalamus. J Comp Neurol. 1997 Nov 3;387(4):588-630. doi: 10.1002/(sici)1096-9861(19971103)387:4<588::aid-cne8>3.0.co;2-z.

Supplementary Material ST1. The full protocols for the 3D-Segmented EPI (A) and FLAIR (B) sequences used for the scanning of the MS patients.

(A)

SIEMENS MAGNETOM Prisma

TA: 6:19 PM: FIX Voxel size: 0.7×0.7×0.7 mmRel. SNR: 1.00 : epfid

Properties

Prio recon	Off
Load images to viewer	On
Inline movie	Off
Auto store images	On
Load images to stamp segments	Off
Load images to graphic segments	Off
Auto open inline display	Off
Auto close inline display	Off
Start measurement without further preparation	Off
Wait for user to start	Off
Start measurements	Single measurement

Routine

Slab group	1
Slabs	1
Dist. factor	50 %
Position	L0.6 P8.4 F14.5 mm
Orientation	Sagittal
Phase enc. dir.	A >> P
AutoAlign	---
Phase oversampling	0 %
Slice oversampling	0.0 %
Slices per slab	256
FoV read	256 mm
FoV phase	87.5 %
Slice thickness	0.67 mm
TR	64 ms
TE	35.0 ms
Averages	1
Concatenations	1
Filter	Raw filter, Prescan Normalize
Coil elements	HE1-4;NE1,2

Contrast - Common

TR	64 ms
TE	35.0 ms
MTC	Off
Flip angle	10 deg
Fat suppr.	Water excit. normal
SWI	Off

Contrast - Dynamic

Averages	1
Averaging mode	Long term
Reconstruction	Magn./Phase
Measurements	1
Multiple series	Each measurement

Resolution - Common

FoV read	256 mm
FoV phase	87.5 %
Slice thickness	0.67 mm
Base resolution	384
Phase resolution	100 %
Slice resolution	100 %
Phase partial Fourier	Off
Slice partial Fourier	Off

Resolution - Common

Interpolation	Off

Resolution - Filter Image

Distortion Corr.	Off
Prescan Normalize	On
Unfiltered images	Off

Resolution - Filter Rawdata

Raw filter	On
Elliptical filter	Off
Hamming	Off

Geometry - Common

Slab group	1
Slabs	1
Dist. factor	50 %
Position	L0.6 P8.4 F14.5 mm
Orientation	Sagittal
Phase enc. dir.	A >> P
Slice oversampling	0.0 %
Slices per slab	256
FoV read	256 mm
FoV phase	87.5 %
Slice thickness	0.67 mm
TR	64 ms
Multi-slice mode	Interleaved
Series	Interleaved
Concatenations	1

Geometry - AutoAlign

Slab group	1
Position	L0.6 P8.4 F14.5 mm
Orientation	Sagittal
Phase enc. dir.	A >> P
AutoAlign	---
Initial Position	L0.6 P8.4 F14.5
L	0.6 mm
P	8.4 mm
F	14.5 mm
Initial Rotation	0.00 deg
Initial Orientation	Sagittal

Geometry - Saturation

Fat suppr.	Water excit. normal
Special sat.	None

Geometry - Tim Planning Suite

Set-n-Go Protocol	Off
Table position	H
Table position	0 mm
Inline Composing	Off

System - Miscellaneous

Positioning mode	FIX
Table position	H
Table position	0 mm
MSMA	S - C - T
Sagittal	R >> L

SIEMENS MAGNETOM Prisma

System - Miscellaneous

Coronal	A >> P
Transversal	F >> H
Coil Combine Mode	Adaptive Combine
Save uncombined	Off
Matrix Optimization	Off
AutoAlign	---
Coil Select Mode	On - AutoCoilSelect

System - Adjustments

B0 Shim mode	Advanced
B1 Shim mode	TrueForm
Adjust with body coil	Off
Confirm freq. adjustment	Off
Assume Dominant Fat	Off
Assume Silicone	Off
Adjustment Tolerance	Auto

System - Adjust Volume

Position	L0.6 P8.4 F14.5 mm
Orientation	Sagittal
Rotation	0.00 deg
A >> P	224 mm
F >> H	256 mm
R >> L	172 mm
Reset	Off

System - pTx Volumes

B1 Shim mode	TrueForm

System - Tx/Rx

Frequency 1H	123.251972 MHz
Correction factor	1
Gain	Low
Img. Scale Cor.	1.000
Reset	Off
? Ref. amplitude 1H	0.000 V

Physio - Signal1

1st Signal/Mode	None
TR	64 ms
Concatenations	1

Sequence - Part 1

Introduction	Off
Dimension	3D
Multi-slice mode	Interleaved
Free echo spacing	Off
Echo spacing	2.76 ms
Bandwidth	394 Hz/Px

Sequence - Part 2

EPI factor	15
RF pulse type	Normal
Gradient mode	Fast
RF spoiling	On

(B)

SIEMENS MAGNETOM Prisma

TA: 5:40 PM: FIX Voxel size: 1.0×1.0×1.0 mmPAT: 2 Rel. SNR: 1.00 : spcir

Properties

Prio recon	Off
Load images to viewer	On
Inline movie	Off
Auto store images	On
Load images to stamp segments	Off
Load images to graphic segments	Off
Auto open inline display	Off
Auto close inline display	Off
Start measurement without further preparation	Off
Wait for user to start	Off
Start measurements	Single measurement

Routine

Slab group	1
Slabs	1
Position	L0.6 P8.4 F14.5 mm
Orientation	Sagittal
Phase enc. dir.	A >> P
AutoAlign	---
Phase oversampling	0 %
Slice oversampling	0.0 %
Slices per slab	176
FoV read	256 mm
FoV phase	93.8 %
Slice thickness	1.00 mm
TR	5000 ms
TE	386 ms
Averages	1.0
Concatenations	1
Filter	Raw filter, Prescan Normalize
Coil elements	HE1-4;NE1,2

Contrast - Common

TR	5000 ms
TE	386 ms
MTC	Off
Magn. preparation	Non-sel. T2-IR
TI 1	1800 ms
Fat suppr.	None
Blood suppr.	Off
Restore magn.	Off

Contrast - Dynamic

Averages	1.0
Reconstruction	Magnitude
Measurements	1
Multiple series	Each measurement

Resolution - Common

FoV read	256 mm
FoV phase	93.8 %
Slice thickness	1.00 mm
Base resolution	256
Phase resolution	100 %
Slice resolution	92 %
Phase partial Fourier	Allowed
Slice partial Fourier	7/8

Resolution - Common

Interpolation	Off

Resolution - iPAT

PAT mode	GRAPPA
Accel. factor PE	2
Ref. lines PE	24
Accel. factor 3D	1
Reference scan mode	Integrated

Resolution - Filter Image

Image Filter	Off
Distortion Corr.	Off
Prescan Normalize	On
Unfiltered images	Off
Normalize	Off
B1 filter	Off

Resolution - Filter Rawdata

Raw filter	On
Elliptical filter	Off

Geometry - Common

Slab group	1
Slabs	1
Position	L0.6 P8.4 F14.5 mm
Orientation	Sagittal
Phase enc. dir.	A >> P
Slice oversampling	0.0 %
Slices per slab	176
FoV read	256 mm
FoV phase	93.8 %
Slice thickness	1.00 mm
TR	5000 ms
Series	Interleaved
Concatenations	1

Geometry - AutoAlign

Slab group	1
Position	L0.6 P8.4 F14.5 mm
Orientation	Sagittal
Phase enc. dir.	A >> P
AutoAlign	---
Initial Position	L0.6 P8.4 F14.5
L	0.6 mm
P	8.4 mm
F	14.5 mm
Initial Rotation	0.00 deg
Initial Orientation	Sagittal

Geometry - Saturation

Fat suppr.	None
Restore magn.	Off
Special sat.	None

Geometry - Navigator

Geometry - Tim Planning Suite

Set-n-Go Protocol	Off

SIEMENS MAGNETOM Prisma

Geometry - Tim Planning Suite

Table position	H
Table position	0 mm
Inline Composing	Off

System - Miscellaneous

Positioning mode	FIX
Table position	H
Table position	0 mm
MSMA	S - C - T
Sagittal	R >> L
Coronal	A >> P
Transversal	F >> H
Coil Combine Mode	Adaptive Combine
Save uncombined	Off
Matrix Optimization	Off
AutoAlign	---
Coil Select Mode	On - AutoCoilSelect

System - Adjustments

B0 Shim mode	Standard
B1 Shim mode	TrueForm
Adjust with body coil	Off
Confirm freq. adjustment	Off
Assume Dominant Fat	Off
Assume Silicone	Off
Adjustment Tolerance	Auto

System - Adjust Volume

Position	L0.6 P8.4 F14.5 mm
Orientation	Sagittal
Rotation	0.00 deg
A >> P	240 mm
F >> H	256 mm
R >> L	176 mm
Reset	Off

System - pTx Volumes

B1 Shim mode	TrueForm
Excitation	Non-sel.

System - Tx/Rx

Frequency 1H	123.251972 MHz
Correction factor	1
Gain	High
Img. Scale Cor.	1.000
Reset	Off
? Ref. amplitude 1H	0.000 V

Physio - Signal1

1st Signal/Mode	None
Trigger delay	0 ms
TR	5000 ms
Concatenations	1

Physio - Cardiac

Magn. preparation	Non-sel. T2-IR
TI 1	1800 ms
Fat suppr.	None
Dark blood	Off
FoV read	256 mm
FoV phase	93.8 %
Phase resolution	100 %

Physio - PACE

Resp. control	Off
Concatenations	1

Inline - Common

Subtract	Off
Measurements	1
StdDev	Off
Save original images	On

Inline - MIP

MIP-Sag	Off
MIP-Cor	Off
MIP-Tra	Off
MIP-Time	Off
Save original images	On

Inline - Composing

Inline Composing	Off
Distortion Corr.	Off

Sequence - Part 1

Introduction	Off
Dimension	3D
Elliptical scanning	Off
Reordering	Linear
Flow comp.	No
Echo spacing	3.42 ms
Adiabatic-mode	Off
Bandwidth	751 Hz/Px

Sequence - Part 2

Echo train duration	858 ms
RF pulse type	Normal
Gradient mode	Fast
Excitation	Non-sel.
Flip angle mode	T2 var
Turbo factor	278

Sequence - Assistant

Allowed delay	180 s

Supplementary Material ST2. The full protocol for the GRE sequences used for the scanning of the healthy control.

SIEMENS MAGNETOM Prisma

TA: 7:02 PM: FIX Voxel size: 0.7×0.7×2.0 mmPAT: 3 Rel. SNR: 1.00 : fl

Properties

Prio recon	Off
Load images to viewer	On
Inline movie	Off
Auto store images	On
Load images to stamp segments	Off
Load images to graphic segments	Off
Auto open inline display	Off
Auto close inline display	Off
Start measurement without further preparation	Off
Wait for user to start	Off
Start measurements	Single measurement

Routine

Slab group	1
Slabs	1
Dist. factor	20 %
Position	R1.2 A16.9 H30.7 mm
Orientation	Transversal
Phase enc. dir.	A >> P
AutoAlign	---
Phase oversampling	0 %
Slice oversampling	0.0 %
Slices per slab	88
FoV read	224 mm
FoV phase	100.0 %
Slice thickness	2.00 mm
TR	39.0 ms
TE	20.00 ms
Averages	1
Concatenations	1
Filter	Raw filter, Prescan Normalize
Coil elements	HC1-7

Contrast - Common

TR	39.0 ms
TE	20.00 ms
MTC	Off
Magn. preparation	None
Flip angle	15 deg
Fat suppr.	None
Water suppr.	None
SWI	Off

Contrast - Dynamic

Averages	1
Averaging mode	Short term
Reconstruction	Magn./Phase
Measurements	1
Multiple series	Each measurement

Resolution - Common

FoV read	224 mm
FoV phase	100.0 %
Slice thickness	2.00 mm
Base resolution	320
Phase resolution	100 %
Slice resolution	100 %
Phase partial Fourier	Off

Resolution - Common

Slice partial Fourier	Off
Interpolation	Off

Resolution - iPAT

PAT mode	GRAPPA
Accel. factor PE	3
Ref. lines PE	24
Accel. factor 3D	1
Reference scan mode	Integrated

Resolution - Filter Image

Image Filter	Off
Distortion Corr.	Off
Prescan Normalize	On
Unfiltered images	On
Normalize	Off
B1 filter	Off

Resolution - Filter Rawdata

Raw filter	On
Elliptical filter	Off

Geometry - Common

Slab group	1
Slabs	1
Dist. factor	20 %
Position	R1.2 A16.9 H30.7 mm
Orientation	Transversal
Phase enc. dir.	A >> P
Slice oversampling	0.0 %
Slices per slab	88
FoV read	224 mm
FoV phase	100.0 %
Slice thickness	2.00 mm
TR	39.0 ms
Multi-slice mode	Interleaved
Series	Interleaved
Concatenations	1

Geometry - AutoAlign

Slab group	1
Position	R1.2 A16.9 H30.7 mm
Orientation	Transversal
Phase enc. dir.	A >> P
AutoAlign	---
Initial Position	R1.2 A16.9 H30.7
R	1.2 mm
A	16.9 mm
H	30.7 mm
Initial Rotation	0.00 deg
Initial Orientation	Transversal

Geometry - Saturation

Saturation mode	Standard
Fat suppr.	None
Water suppr.	None
Special sat.	None

Geometry - Tim Planning Suite

Set-n-Go Protocol	Off

SIEMENS MAGNETOM Prisma

Geometry - Tim Planning Suite

Table position	H
Table position	0 mm
Inline Composing	Off

System - Miscellaneous

Positioning mode	FIX
Table position	H
Table position	0 mm
MSMA	S - C - T
Sagittal	R >> L
Coronal	A >> P
Transversal	F >> H
Coil Combine Mode	Adaptive Combine
Save uncombined	Off
Matrix Optimization	Off
AutoAlign	---
Coil Select Mode	On - AutoCoilSelect

System - Adjustments

B0 Shim mode	Standard
B1 Shim mode	TrueForm
Adjust with body coil	Off
Confirm freq. adjustment	Off
Assume Dominant Fat	Off
Assume Silicone	Off
Adjustment Tolerance	Auto

System - Adjust Volume

! Position	R1.2 A16.9 H30.7 mm
! Orientation	Transversal
! Rotation	0.00 deg
! A >> P	224 mm
! R >> L	224 mm
! F >> H	176 mm
Reset	Off

System - pTx Volumes

B1 Shim mode	TrueForm
Excitation	Slab-sel.

System - Tx/Rx

Frequency 1H	123.248991 MHz
Correction factor	1
Gain	Low
Img. Scale Cor.	1.000
Reset	Off
? Ref. amplitude 1H	0.000 V

Physio - Signal1

1st Signal/Mode	None
TR	39.0 ms
Concatenations	1
Segments	1

Physio - Cardiac

Tagging	None
Magn. preparation	None
Fat suppr.	None
Dark blood	Off
FoV read	224 mm
FoV phase	100.0 %
Phase resolution	100 %

Physio - PACE

Resp. control	Off
Concatenations	1

Inline - Common

Subtract	Off
Measurements	1
StdDev	Off
Liver registration	Off
Save original images	On

Inline - MIP

MIP-Sag	Off
MIP-Cor	Off
MIP-Tra	Off
MIP-Time	Off
Save original images	On

Inline - Soft Tissue

Wash - In	Off
Wash - Out	Off
TTP	Off
PEI	Off
MIP - time	Off
Measurements	1

Inline - Composing

Inline Composing	Off
Distortion Corr.	Off

Sequence - Part 1

Introduction	Off
Dimension	3D
Elliptical scanning	Off
Phase stabilisation	Off
Asymmetric echo	Off
Contrasts	1
Flow comp.	No
Multi-slice mode	Interleaved
Bandwidth	300 Hz/Px

Sequence - Part 2

Segments	1
Acoustic noise reduction	None
RF pulse type	Normal
Gradient mode	Fast
Excitation	Slab-sel.
RF spoiling	On

Sequence - Assistant

Mode	Off
Allowed delay	0 s

Supplementary Material ST3. The full protocol for the single-average 3D-Segmented EPI sequences used for the scanning of the healthy control. The same sequence with the exact same parameters (*except Averages: 2*) was used for the 2-averages 3D-Segmented EPI.

TA: 1:56 PM: FIX Voxel size: 0.7×0.7×2.0 mmPAT: 3 Rel. SNR: 1.00 : epfid

Properties

Prio recon	Off
Load images to viewer	On
Inline movie	Off
Auto store images	On
Load images to stamp segments	Off
Load images to graphic segments	Off
Auto open inline display	Off
Auto close inline display	Off
Start measurement without further preparation	Off
Wait for user to start	Off
Start measurements	Single measurement

Routine

Slab group	1
Slabs	1
Dist. factor	50 %
Position	R1.2 A16.9 H30.7 mm
Orientation	Transversal
Phase enc. dir.	A >> P
AutoAlign	---
Phase oversampling	0 %
Slice oversampling	0.0 %
Slices per slab	88
FoV read	224 mm
FoV phase	100.0 %
Slice thickness	2.00 mm
TR	56 ms
TE	20.0 ms
Averages	1
Concatenations	1
Filter	Raw filter, Prescan Normalize
Coil elements	HC1-7

Contrast - Common

TR	56 ms
TE	20.0 ms
MTC	Off
Flip angle	21 deg
Fat suppr.	Water excit. normal
SWI	Off

Contrast - Dynamic

Averages	1
Averaging mode	Long term
Reconstruction	Magn./Phase
Measurements	1
Multiple series	Each measurement

Resolution - Common

FoV read	224 mm
FoV phase	100.0 %
Slice thickness	2.00 mm
Base resolution	320
Phase resolution	100 %
Slice resolution	100 %
Phase partial Fourier	Off
Slice partial Fourier	Off
Interpolation	Off

Resolution - iPAT

PAT mode	GRAPPA
Accel. factor PE	3
Ref. lines PE	24
Accel. factor 3D	1
Ref. lines 3D	24
Reference scan mode	GRE/separate

Resolution - Filter Image

Distortion Corr.	Off
Prescan Normalize	On
Unfiltered images	On

Resolution - Filter Rawdata

Raw filter	On
Elliptical filter	Off
Hamming	Off

Geometry - Common

Slab group	1
Slabs	1
Dist. factor	50 %
Position	R1.2 A16.9 H30.7 mm
Orientation	Transversal
Phase enc. dir.	A >> P
Slice oversampling	0.0 %
Slices per slab	88
FoV read	224 mm
FoV phase	100.0 %
Slice thickness	2.00 mm
TR	56 ms
Multi-slice mode	Interleaved
Series	Interleaved
Concatenations	1

Geometry - AutoAlign

Slab group	1
Position	R1.2 A16.9 H30.7 mm
Orientation	Transversal
Phase enc. dir.	A >> P
AutoAlign	---
Initial Position	R1.2 A16.9 H30.7
R	1.2 mm
A	16.9 mm
H	30.7 mm
Initial Rotation	0.00 deg
Initial Orientation	Transversal

Geometry - Saturation

Fat suppr.	Water excit. normal
Special sat.	None

Geometry - Tim Planning Suite

Set-n-Go Protocol	Off
Table position	H
Table position	0 mm
Inline Composing	Off

System - Miscellaneous

Positioning mode	FIX
Table position	H

SIEMENS MAGNETOM Prisma

System - Miscellaneous

Table position	0 mm
MSMA	S - C - T
Sagittal	R >> L
Coronal	A >> P
Transversal	F >> H
Coil Combine Mode	Adaptive Combine
Save uncombined	Off
Matrix Optimization	Off
AutoAlign	---
Coil Select Mode	On - AutoCoilSelect

System - Adjustments

B0 Shim mode	Standard
B1 Shim mode	TrueForm
Adjust with body coil	Off
Confirm freq. adjustment	Off
Assume Dominant Fat	Off
Assume Silicone	Off
Adjustment Tolerance	Auto

System - Adjust Volume

Position	R1.2 A16.9 H30.7 mm
Orientation	Transversal
Rotation	0.00 deg
A >> P	224 mm
R >> L	224 mm
F >> H	176 mm
Reset	Off

System - pTx Volumes

B1 Shim mode	TrueForm

System - Tx/Rx

Frequency 1H	123.248991 MHz
Correction factor	1
Gain	Low
Img. Scale Cor.	1.000
Reset	Off
? Ref. amplitude 1H	0.000 V

Physio - Signal1

1st Signal/Mode	None
TR	56 ms
Concatenations	1

Sequence - Part 1

Introduction	Off
Dimension	3D
Reordering	Linear
Multi-slice mode	Interleaved
Free echo spacing	Off
Echo spacing	2.75 ms
Bandwidth	422 Hz/Px

Sequence - Part 2

EPI factor	5
RF pulse type	Normal
Gradient mode	Fast
RF spoiling	On

Supplementary Material ST4. ROI-assessed QSM mean and standard deviation (Mean ± SD, in ppb) for all reconstructions (control subject).

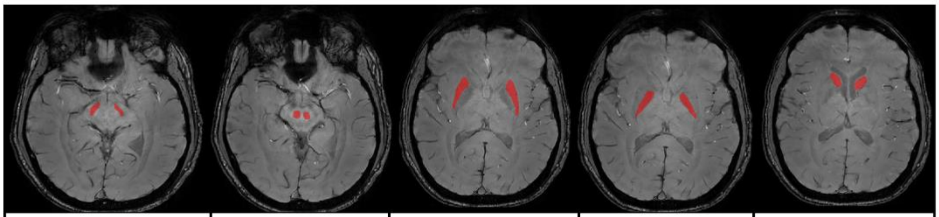

		Substantia Nigra	Red Nucleus	Putamen	Pallidum	Caudate
CR	GRE	87.0 ± 44.0	76.2 ± 25.9	22.9 ± 24.8	80.7 ± 25.5	17.1 ± 16.8
	3DEPI2	83.9 ± 40.3	70.1 ± 26.0	21.8 ± 25.0	81.2 ± 29.6	15.0 ± 20.0
	3DEPI1	84.4 ± 41.5	71.9 ± 27.8	23.6 ± 24.8	79.3 ± 31.3	14.7 ± 20.3
DLR	GRE	84.7 ± 40.5	74.8 ± 23.1	22.6 ± 24.3	79.7 ± 24.9	17.0 ± 16.3
	3DEPI2	82.6 ± 37.6	71.4 ± 25.0	21.7 ± 24.1	80.5 ± 29.5	16.1 ± 20.0
	3DEPI1	81.8 ± 38.8	72.1 ± 26.4	21.8 ± 24.2	80.4 ± 31.0	15.9 ± 19.5

Supplementary Material ST5. Sensitivity and specificity metrics for both raters were assessed in classifying multiple sclerosis (MS) lesion characteristics using DLR and CR-based QSM. Ground truth was established through consensus: initial agreement between the two raters was confirmed or adjudicated by a third independent rater in cases of disagreement. Metrics are reported separately for paramagnetic rim lesions (PRLs), hyper/isointense lesions, central vein sign (CVS) identification, and for all MS lesions combined. CR-QSM denotes the conventionally reconstructed 3DEPI-based χ-map and the DLR-QSM denotes the deep Learning- reconstructed 3DEPI-based χ-map.

**Supplementary Table 5:** Sensitivity and Specificity		**Rater 1**		**Rater 2**	
		**CR-QSM**	**DLR-QSM**	**CR-QSM**	**DLR-QSM**
**Sensitivity**	**Paramagnetic Rim Lesions**	0.97	0.97	1.00	0.99
	**Hyper/Isointense QSM Lesions**	0.76	0.76	0.93	0.94
	**Central Vein Sign**	0.62	0.62	0.82	0.85
	**All Lesions**	0.75	0.75	0.91	0.92
**Specificity**	**Paramagnetic Rim Lesions**	1.00	1.00	0.99	1.00
	**Hyper/Isointense QSM Lesions**	0.98	0.98	0.96	0.97
	**Central Vein Sign**	0.99	0.99	0.97	0.97
	**All Lesions**	0.99	0.99	0.97	0.98
